# Origin of Antibiotics and Antibiotic Resistance, and Their Impacts on Drug Development: A Narrative Review

**DOI:** 10.3390/ph16111615

**Published:** 2023-11-15

**Authors:** Ghazala Muteeb, Md Tabish Rehman, Moayad Shahwan, Mohammad Aatif

**Affiliations:** 1 Department of Nursing, College of Applied Medical Science, King Faisal University, Al-Ahsa 31982, Saudi Arabia; 2 Department of Pharmacognosy, College of Pharmacy, King Saud University, Riyadh 11437, Saudi Arabia; mrehman@ksu.edu.sa; 3 Center for Medical and Bio-Allied Health Sciences Research, Ajman University, Ajman 346, United Arab Emirates; m.shahwan@ajman.ac.ae; 4 Department of Clinical Sciences, College of Pharmacy and Health Sciences, Ajman University, Ajman 346, United Arab Emirates; 5 Department of Public Health, College of Applied Medical Sciences, King Faisal University, Al-Ahsa 31982, Saudi Arabia; maahmad@kfu.edu.sa

**Keywords:** antibiotic resistance, drug designing, bacterial mutation, bacterial evolution, horizontal gene transfer, public and agricultural health

## Abstract

Antibiotics have revolutionized medicine, saving countless lives since their discovery in the early 20th century. However, the origin of antibiotics is now overshadowed by the alarming rise in antibiotic resistance. This global crisis stems from the relentless adaptability of microorganisms, driven by misuse and overuse of antibiotics. This article explores the origin of antibiotics and the subsequent emergence of antibiotic resistance. It delves into the mechanisms employed by bacteria to develop resistance, highlighting the dire consequences of drug resistance, including compromised patient care, increased mortality rates, and escalating healthcare costs. The article elucidates the latest strategies against drug-resistant microorganisms, encompassing innovative approaches such as phage therapy, CRISPR-Cas9 technology, and the exploration of natural compounds. Moreover, it examines the profound impact of antibiotic resistance on drug development, rendering the pursuit of new antibiotics economically challenging. The limitations and challenges in developing novel antibiotics are discussed, along with hurdles in the regulatory process that hinder progress in this critical field. Proposals for modifying the regulatory process to facilitate antibiotic development are presented. The withdrawal of major pharmaceutical firms from antibiotic research is examined, along with potential strategies to re-engage their interest. The article also outlines initiatives to overcome economic challenges and incentivize antibiotic development, emphasizing international collaborations and partnerships. Finally, the article sheds light on government-led initiatives against antibiotic resistance, with a specific focus on the Middle East. It discusses the proactive measures taken by governments in the region, such as Saudi Arabia and the United Arab Emirates, to combat this global threat. In the face of antibiotic resistance, a multifaceted approach is imperative. This article provides valuable insights into the complex landscape of antibiotic development, regulatory challenges, and collaborative efforts required to ensure a future where antibiotics remain effective tools in safeguarding public health.

## 1. Introduction

Among the greatest discoveries of humankind in the 20th century was the discovery of antibiotics. Antibacterial triumph changed contemporary biomedicine and seeks to define, mold, and grow both its potential and its boundaries. Regrettably, the possibility for resistance to any therapeutic agent to evolve limits its ability to be effective [1,2]. The next series of antibiotics must be developed, since resistance compromises efficacy (therapeutic effect). A pathogen’s enhanced resistance to the prescribed standard therapy to which it was previously vulnerable is referred to as tolerance to an antibacterial agent (in this case, an antibiotic) [3,4,5].

The history of using antimicrobial agents to combat infections is rich, dating back to ancient civilizations where various natural extracts were employed for their healing properties. Some of these extracts, originating from plants and molds, exhibited antibacterial properties, even before the term “antibiotics” was coined [6]. The introduction of the term “antibiotics” was the result of pioneering work by American microbiologist Selman Waksman and his team, who successfully isolated chemical substances from microorganisms capable of inhibiting the growth of other microbes [7]. While the concept of using microorganisms to combat infections has ancient roots, it was Alexander Fleming’s serendipitous discovery of penicillin in 1928 that marked the inception of modern antibiotic therapy [8]. Fleming’s discovery bridged the gap between ancient knowledge, such as the Egyptians’ use of moldy bread to treat infection, and the era of antibiotics [8]. The post-World War II period, often referred to as the “golden era” of antibiotic discovery, witnessed the identification of numerous antibiotic classes that continue to be used today [9]. The advent of penicillin rapidly propagated the belief that infections could be effectively controlled with antibiotics, despite earlier use of sulfonamides as the first antimicrobials, which faced limitations due to emerging resistance mechanisms that persist to this day [10]. Interestingly, the penicillin discovery team identified penicillinase, a bacterium capable of degrading penicillin, even before widespread access to the antibiotic [11,12]. Subsequent decades brought forth remarkable progress, marked by the development of antibiotics like streptomycin, chloramphenicol, tetracyclines, erythromycin, vancomycin, cephalosporins, and others. This expansion of the antibiotic arsenal made previously fatal diseases treatable, cementing the antibiotic age [13,14]. The post-World War II era also saw the emergence of semi-synthetic antibiotics like amoxicillin and quinolones, notable for their enhanced stability and broader antibacterial spectra. Antibiotics such as vancomycin played pivotal roles in combating drug-resistant bacterial strains, particularly methicillin-resistant Staphylococcus aureus (MRSA). Innovations continued with the development of macrolides, third-generation cephalosporins, daptomycin, and linezolid, addressing Gram-negative resistance and enhancing antibiotic pharmacokinetics [15,16]. However, despite these advancements, antibiotic-resistant bacterial strains have proliferated in recent decades [17,18], leading to a reassessment of antibiotic usage, increased awareness of antibiotic resistance, and the implementation of antibiotic stewardship programs, alongside the exploration of novel strategies like phage therapy, combination therapies, and precision-medicine approaches to combat drug-resistant bacteria [19,20].

The global scenario regarding antibiotic resistance remains a pressing issue for public health, with a consistent upward trend in resistance prevalence over the past few decades. This phenomenon extends its reach to a broad spectrum of bacteria, rendering many antibiotics less effective or entirely impotent against infections. Consequently, once easily treatable common infections have become more formidable adversaries, resulting in prolonged hospitalizations, elevated healthcare expenses, and heightened mortality rates. One of the paramount challenges associated with antibiotic resistance is the sluggish pace of new antibiotic development. Several pharmaceutical companies have withdrawn from antibiotic research and development, due to the low profitability of these drugs. Crafting a novel antibiotic is a protracted and costly endeavor, which has led to diminished enthusiasm for innovation in this vital medical domain. The unwarranted and incorrect use of antibiotics continues to be a substantial contributor to resistance development. Antibiotics are frequently prescribed when unnecessary, both in healthcare settings and for common community illnesses. Furthermore, the use of antibiotics in agriculture and animal husbandry exacerbates resistance dissemination, particularly through practices such as using antibiotics as growth promoters in livestock, potentially transmitting resistance to humans through the food chain.

Efforts to combat antibiotic resistance have gained momentum through global initiatives and organizations like the World Health Organization (WHO). These entities work diligently to heighten awareness about antibiotic resistance, champion responsible antibiotic use, and advocate for innovative antibiotic research. The One Health approach, acknowledging the interconnectedness of human, animal, and environmental health, has gained traction as a comprehensive strategy for addressing antibiotic resistance. This approach recognizes that the application of antibiotics in any one sector can have repercussions on resistance in other sectors. Notably, regional variations exist in antibiotic resistance rates and trends, contingent on dissimilar healthcare practices, antibiotic usage patterns, and regulatory frameworks. In summary, antibiotic resistance remains a formidable global public health concern. While significant strides have been made in raising awareness and promoting responsible antibiotic use, the inexorable ascent of antibiotic resistance and the dearth of new antibiotic development underscore the imperative need for continuous efforts to combat this critical issue. The collaborative endeavors of healthcare professionals, researchers, policymakers, and the public remain pivotal to mitigating the impact of antibiotic resistance on global health.

The primary objectives and aims of this comprehensive review article are to address the global challenge of antibiotic resistance, explore innovative strategies against drug-resistant microorganisms, examine economic challenges in antibiotic development, and propose strategies to re-engage pharmaceutical firms. It emphasizes the importance of international collaborations and government-led initiatives in combating antibiotic resistance.

## 2. Origin of Antibiotic Resistance

The survivability of pathogens against the exposure and administration of antibiotics that could kill them or restrict their proliferation is called antibiotic resistance. There are several factors behind the expression of antibiotic resistance against antibiotics or antimicrobial agents, including the degree of resistance expression of the bacterial strain and its ability to survive through resistance mechanisms [21,22]. Microbial strains may possess inherent resistance or react strongly to transgene expression from one bacterium to another via plasmids, transgenes, genetic elements, and phages, or through alterations in cell genes (chromatin instability) that result in cross-resistance [23]. Resistant microorganisms may proliferate swiftly if resistance factors are present on plasmids. Biochemical mechanisms work behind the resistance to protect the bacterial cell wall from various agents, which trigger target alteration, enzymatic degradation, and reduced or increased uptake of efflux pump proteins. Thus, first-generation antibiotics have been facing antibiotic resistance in various clinical practices through natural processes [24].

Antibiotic resistance has become a global challenge for human health, as it is linked to high rates of mortality and morbidity. The Gram-positive and Gram-negative bacteria-related infections became difficult to treat due to multidrug resistance, and could not be treated with traditional antibiotics. In both pre-antibiotic and antibiotic periods, antibiotic resistance has badly affected antibiotic efficacy in clinical practices [25]. The first antibiotic resistance was reported as soon as sulfonamides were introduced in 1930; it predicted the occurrence of antibiotic resistance in the natural environment in the pre-antibiotic era without supporting the existence of deadly resistant pathogens. The advancement of antibiotics and their application in other aspects of life led to the beginning of the antibiotic era. As an outcome of human activity, high concentrations of toxic doses of antibiotics were used, which caused a significant alteration in their inherent significance and the rapid growth of bacteria that were antibiotic-resistant. 

Antibiotic resistance is as ancient as the clinical use of antibiotics, as it has become prevalent and resistant due to the capability of drug agents acting to inactivate their cell walls. Research evidence suggests the chemical modification of antibiotics to restrict or limit the cleavage by penicillinases (β-lactamases) [26]. However, the detection and inactivation of bacterial penicillinase can enhance the efficacy of antibiotics, and these are genes or components of other useful microorganisms, as the different research findings reported. Increased use of antibiotics, genetic modification in bacterial strains, and antibiotic resistance genes have been involved in the cellular mechanism of antibiotic resistance before human intervention. They have also been released from natural genomic sources, rapidly spreading to infectious and commensal bacteria with different taxonomic classifications. 

The total absence of viable preventative measures, the difficulty in dealing with bacterial infections and their accompanying diseases, and the small number of new antibacterial drugs in progress in the clinic would then necessitate the advancement of novel treatment approaches and broad-spectrum antimicrobial treatments. In patients with bacteremia and other serious illnesses, the rapid recognition of pathogenic bacteria and their antimicrobial sensitivity trends is lacking. According to the researchers, a greater knowledge of how infectious disease-induced biochemical mechanisms and specific virulence strategies work offers new opportunities to attack and interact with critical pathogenicity variables or infectivity characteristics of bacteria without subjecting them to pressure from evolution to acquire resistance.

## 3. Mechanism of Antibiotic Resistance

**Figure 1:**
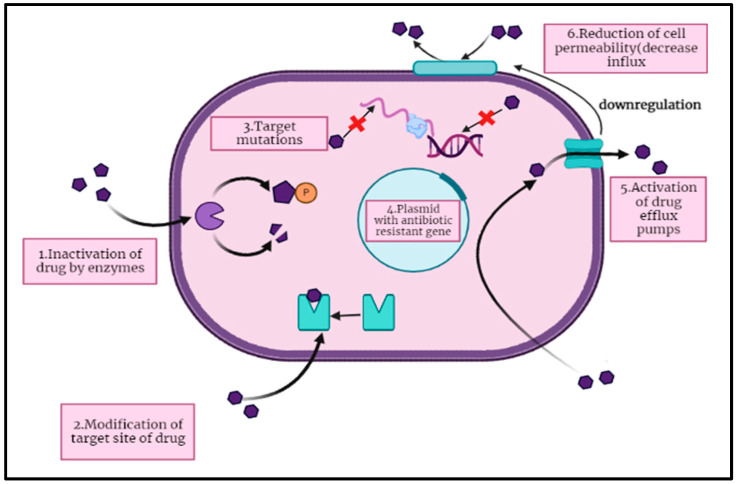
Different pathways of antibiotic resistance as antibiotic enters into the cell of bacteria or microorganisms.

The antibiotics mainly attack the biochemistry and physiology of the microbial cells to reduce their growth or cause death. Some antibiotics destroy the cell walls or cell membranes of bacterial cells by dissolving the β-lactam and glycopeptide components, while other antibiotics target the protein synthetic machinery by linking with ribosomal units, which stops the antibacterial activity of those microbes (Figure 1) [18,27,28,29]. These cell-wall-targeting antibiotics include aminoglycosides, tetracycline, linezolid, chloramphenicol, and macrolides. The other cell-machinery-targeting antibiotics and nucleic acid synthesis interfering molecules include rifampin and fluoroquinolones (FQ). The remaining antibiotics are those that interfere with metabolic pathways and destroy the membrane matrix, including folic acid analogs, daptomycin, polymyxins, and sulfonamides [30].

The research evidence suggests that the evolution of antibiotic resistance determinants induces modification among other antibiotic-sensitive counterparts upon antibiotic exposure. Among bacterial strains, multidrug resistance or related determinants induce resistance for other chemically unrelated compounds rather than antibiotics, and those include quaternary ammonium compounds [31], the anionic detergent sodium dodecyl sulfate, ethidium bromide, the DNA-intercalating mutagen acridine, and uncouplers. Therefore, multidrug resistance has a greater impact on bacterial physiology. Additionally, they provide resistance to various metabolic products made by the organism, such as bile acids. It is hypothesized that some as-yet-unidentified biological functions of the factors in these bacteria lead to resistance to antibiotics [32].

**Figure 2:**
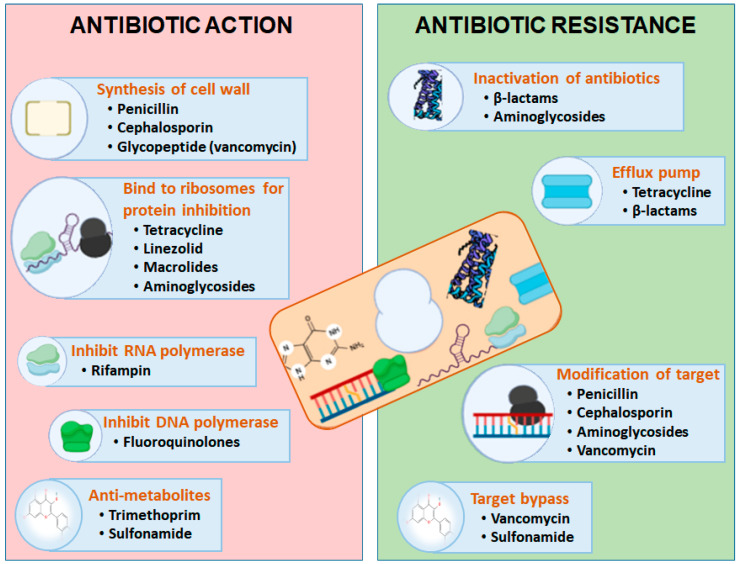
Differentiating the antibiotic action and antibiotic resistance through different aspects within a bacterial cell.

**Genetic mutations:** Antibiotic resistance also emerges through the genetic mutations encoded on the bacterial chromosome, which occur due to the evolution of genetic material against antibiotics and its transfer to the next generation (Figure 2). Genetic mutations can occur through five mechanisms: substitution, deletion, addition, inversion, and duplication. For example, spontaneous resistance to fluoroquinolones like ciprofloxacin and norfloxacin can develop due to mutations in the DNA gyrase coding genes gyrA and gyrB [33,34]. Similarly, resistance of pathogenic bacteria to macrolides (such as erythromycin, azithromycin, clarithromycin, dirithromycin, troleandomycin, etc.) may result from mutational changes in specific nucleotides of the ribosomal RNA (rRNA) 23S sequence [35,36]. Another mechanism of resistance includes plasmid transfer, and that is likely the most crucial; it can end up supplying the host organism and its offspring with additional genetic information expressing antibiotic resistance. Antibiotics have an impact on this by causing the transmission of resistant genes among microbes as well as by applying selection pressure towards the development of resistant bacteria. Other antibiotic-resistant strains transfer the genes encoding resistance for particular antibiotics, which evolve through mutation [37,38] and those genes are associated with the antibiotic biosynthesis pathway either in clusters or as single genes. As evidence from *Streptomyces coelicolor* predicted, the degree of antibiotic resistance is linked with the antibiotic biosynthetic gene as it determines the role of antibiotic resistance determinants and postulates about the biosynthetic pathway of antibiotics [39]. Even in isolates that do not generate antibiotic-resistant genes, they are usually present. As a result, bacteria have developed a wide range of genes (known as the “resistome”) that shield them from the effective dose of antibiotics. Clinically relevant resistant infections contain mobility genetic elements with genes orthologous to these on them. There is a chance that the genes that help compensate for this external resistance will spread to pathogens, and there is some proof at least that many significant clinical gene mutations have their roots in environmental microorganisms [40].

**Mutation of Target Sites:** These mutations can also affect the target sites of antibiotics, such as enzymes that the antibiotic interferes with or the bacterial ribosomes that the antibiotic binds to [41]. Mutations in these target sites can alter the structure of the binding site, reducing the affinity of the antibiotic for its target and rendering it less effective. As a result, the bacterium becomes less susceptible to the antibiotic’s action, leading to resistance. Additionally, mutations can also upregulate efflux pumps—specialized proteins that actively pump antibiotics out of the bacterial cell before they can exert their effects. For instance, mutations in the β-subunit of RNA polymerase gene of *Mycobacterium tuberculosis* can lead to resistance against the antibiotic rifampicin [42]. This antibiotic normally inhibits bacterial RNA synthesis by binding to the bacterial RNA polymerase. Mutations in the RNA polymerase gene alter the structure of the binding site, reducing the affinity of rifampicin for the target and preventing its effective inhibition [43].

**Epigenetic mechanisms:** The antibiotic resistance genes and their epigenetic inheritance-based evolution have been found first in isolated strains of *Escherichia coli*, which have been exposed to different concentrations of antibiotics including tetracycline, nalidixic acid, and ampicillin [44,45]. Another study that used the data of de novo assembly of short-read sequences in conjugation with a metagenomic approach suggested that lateral transfer of antibiotic resistance genes with a non-coding region occurs due to the mobilization of modified sequences among clinical pathogens from the environmental reservoir of soil bacteria. Randomized uncontrolled evolution really cannot explain the high probability of survival on lower antimicrobial agents. It was instead proposed that the genes for resistance to antibiotics were obtained through epigenetic mechanisms. Because of the maintenance of specific chromatin configurations or DNA methylation states, the high rate of reversal of these resistive phenotypes further demonstrated that it was an instance of epigenetic mechanisms, which do not confer a durable phenotype [46]. 

**Horizontal gene transfer:** The lateral gene transfer or horizontal gene transfer (HGT) contributes to the transfer of resistivity, its evolution, and maintenance among pathogenic bacteria and also plays a role in the destruction of antibiotic resistance genes (ARGs) transferred from the natural environment in clinical settings [47]. The best example of HGT is the spread of carbapenem resistance in Enterobacteriaceae. The blaNDM-1 gene, which encodes a carbapenemase enzyme that breaks down carbapenem antibiotics, can spread rapidly through horizontal gene transfer. This gene has been found in various Enterobacteriaceae species, allowing them to resist the effects of carbapenem antibiotics. The spread of this gene among different bacterial species contributes to the widespread resistance to these last-resort antibiotics. An additional instance of horizontal gene transfer (HGT) is evident in the *Shigella* outbreak within the United Kingdom, resulting from the transfer of a plasmid-borne antibiotic resistance gene [48]. This dissemination of a plasmid carrying resistance to azithromycin enabled the proliferation of pathogens that were once infrequent, due to the decreased effectiveness of conventional antibiotics. In the end, several outbreaks involving distinct strains emerged as a consequence of these strains independently acquiring the same plasmid [49]. 

**Conjugation:** Conjugation, which is one of the mechanism of HGT, is believed to have the biggest impact on the spread of ARGs among the three recognized canonical mechanisms of HGT. Conjugation is the mechanism by which DNA is transferred from cell to cell via cell surface pili or adhesins. It is aided by conjugative machinery, which is encoded by genes on autonomously replicating plasmids or integrative conjugative elements in the chromosome. The challenges of antibiotic resistance (AR) in hospital settings are further complicated by horizontal gene transfer (HGT), and this is prominently illustrated in the context of plasmid-mediated resistance to β-lactam antibiotics. Resistance mechanisms involving extended-spectrum β-lactamases (ESBLs) and carbapenemase, for instance, operate by breaking down β-lactam antibiotics like penicillin, carbapenems, and cephalosporins [50]. Notably, β-lactam resistance genes are frequently situated on plasmids, leading to their dissemination through conjugation within and between different species, particularly within the *Enterobacteriaceae*, *Pseudomonas*, and *Acinetobacter* families [51,52,53,54,55]. 

**Transformation and transduction:** While transformation and transduction (two other mechanisms of HGT) are assumed to be less crucial, recent studies indicate that their involvement may be more substantial than previously thought [56]. Currently, there is only indirect evidence of transduction occurring in hospital settings. For instance, in the case of hospital-acquired MRSA infections, bacteriophages extracted from these infections were observed to efficiently transduce antibiotic resistance genes (ARGs) to susceptible strains under laboratory conditions (as reported in [57]). Similarly, instances of transduction between hospital isolates of *S. aureus* have been documented, where tetracycline and penicillin resistance genes were transferred [58]. In Gram-negative bacteria, laboratory studies have demonstrated the transference of multiple ARGs, including extended-spectrum β-lactamase (ESBL) genes, from *Pseudomonas* hospital isolates to other *Pseudomonas* strains [59]. Additionally, β-lactamase genes have been shown to undergo transduction between *Acinetobacter* strains in controlled settings [60]. It is worth noting that the CTX-M family of ESBL genes, typically carried on plasmids, is believed to have originated in *Kluyvera*, a relatively infrequent pathogen within the Enterobacteriaceae family (10.1086/322686). The horizontal transfer of ESBL genes from minor pathogens like *Kluyvera* has led to the extensive spread of CTX-M variants among clinically significant *E. coli* and *Klebsiella* strains [61,62]. Certain bacteria appeared to be capable of uptake, integration, and functional expression of naked extracellular DNA fragments, a process known as (natural) transformation. Several antibiotic-resistant pathogens with clinical relevance possess the ability to uptake DNA and undergo natural transformation. These include *Acinetobacter*, *Haemophilus*, *Neisseria*, *Pseudomonas*, *Staphylococcus*, and *Streptococcus*, as detailed in References [63,64]. Frederick Griffith’s groundbreaking work demonstrated that nonvirulent Streptococcus pneumoniae strains could transform into virulent pathogens within infected mice, as reported in [65]. Similarly, research has shown that *Helicobacter pylori* can acquire genes through natural competence and undergo transformation, both in colonized humans and in mouse infection models, as documented in [66,67].

Bacteriophages have a significant impact on the bacterial microbiome in any setting. Bacteriophages can transfer genes that are beneficial to their microbial hosts via specialized or generalized transduction, so enhancing their own survival and spread [68]. The mobilization or transfer of ARGs by bacteriophages has been described for a variety of bacterial species, including the transduction of erythromycin, tetracycline, or multiple resistances across *S. pyogenes* strains and the transfer of antibiotic resistance plasmids in MRSA [69]. 

**Biofilm Formation:** In some cases, the antibiotic resistance and tolerance of bacteria to certain antibiotics depends on their metabolic state. This phase of the bacterium also influences its susceptibility to antibiotics, with sometimes high resistivity exhibited by the stationary phase as compared to the growing phase. The production of biofilm in the micro environment during the stationary phase can contribute to the resistance towards antibiotics. Biofilms are structured communities of bacteria embedded within a self-produced extracellular matrix. Biofilm formation enables bacteria to adhere to surfaces and protect themselves from antibiotics and the immune system. Within a biofilm, bacteria can communicate through a process called quorum sensing, allowing them to coordinate their resistance mechanisms. This can lead to higher antibiotic tolerance than individual planktonic (free-floating) cells. The protective matrix of the biofilm can hinder the penetration of antibiotics, making it difficult for drugs to reach and kill the bacteria within. Biofilm generation is especially concerning in clinical settings, since it impedes antibiotic treatment of infections due to the facilitated acquisition of antibiotic resistance genes (ARGs). One such example is chronic *Pseudomonas aeruginosa* infections in cystic fibrosis patients, wherein *Pseudomonas aeruginosa* often forms biofilms in the airways of patients. Within the biofilm, *P. aeruginosa* cells exhibit increased resistance to antibiotics, as the biofilm matrix prevents antibiotics from effectively penetrating and reaching the bacterial cells, allowing the infection to persist and become chronic despite treatment. The intercellular signaling mechanism within *Pseudomonas aeruginosa* regulates the expression of genes associated with superoxide dismutase and catalase, ultimately influencing resistance to hydrogen peroxides [70]. Environmental factors are now recognized as critical in driving biofilm formation, antibiotic resistance development, and spread. Several studies have shown that environmental biofilms might act as hotspots for the spread of ARGs [71]. The reduction in nitrogen production also triggers resistance to antibiotics, which is also called swarming [32]. The swarming cells of bacteria posed resistance to a variety of antibiotics. These data, along with the susceptibility seen in biofilm, strongly suggest that antimicrobial resistance is a result of bacterial social behavior [72,73]. 

**Stringent Response:** Antimicrobial resistance modulation by alarmone (p) ppGpp formation is a sophisticated regulatory mechanism employed by bacteria to adapt and survive under stressful conditions, including exposure to antibiotics. The molecule (p) ppGpp, commonly referred to as “magic spot” or “magic spot molecule,” plays a crucial role in regulating bacterial responses to various stresses, including nutrient limitation and antibiotic exposure [74]. (p) ppGpp is synthesized from GTP (guanosine triphosphate) and ATP (adenosine triphosphate) by enzymes known as RelA and SpoT (or RelQ), collectively referred to as “relA-spoT homologs.” This synthesis occurs when bacteria sense a decrease in the availability of amino acids and other nutrients. The accumulation of (p) ppGpp triggers a series of responses that allow bacteria to prioritize survival and stress adaptation over normal growth and replication. It modulates antibiotic resistance by different mechanisms such as downregulation of growth, stress adaptation, altered gene expression, and biofilm formation. One of the main effects of elevated (p) ppGpp levels is the downregulation of genes involved in rapid growth and replication. When bacteria encounter stressors like antibiotics, they divert their resources away from growth and focus on conserving energy. This reduction in growth can slow down the rate of cell division, making bacteria less susceptible to antibiotics that target actively dividing cells. Elevated ppGpp levels lead to the activation of specific genes that enhance stress adaptation such as activation of efflux pumps which actively pump antibiotics out of bacterial cells, reducing their intracellular concentration and effectiveness.

**Enzymatic Inactivation:** Some bacteria produce enzymes that can chemically modify or degrade antibiotics. For instance, β-lactamase enzymes are capable of breaking down β-lactam antibiotics like penicillin and cephalosporins. By doing so, the antibiotic is inactivated before it can exert its antimicrobial effects. 

**Efflux Pumps:** Bacterial cells can employ efflux pumps, which are membrane proteins, to actively pump antibiotics out of the cell. This reduces the intracellular concentration of the antibiotic, making it less effective. Efflux pumps can expel a wide range of antibiotics and are often responsible for multi-drug resistance. For example, *Pseudomonas aeruginosa*, an opportunistic pathogen, has efflux pumps like MexAB-OprM, which can pump out antibiotics like fluoroquinolones, tetracyclines, and aminoglycosides. This efflux mechanism contributes to the bacterium’s resistance to multiple drug classes.

**Alternative Metabolic Pathways:** Some bacteria can circumvent the effects of antibiotics by utilizing alternative metabolic pathways. For example, instead of utilizing the dihydrofolate reductase targeted by trimethoprim, bacteria can use a different enzyme, rendering the antibiotic ineffective. 

**Altered Permeability:** Changes in the permeability of the bacterial cell membrane can influence how antibiotics enter the cell. Reduced permeability can reduce antibiotic uptake and thus effectiveness. Example: Gram-negative bacteria like *Escherichia coli* can become resistant to tetracycline antibiotics by decreasing the permeability of their outer membrane, preventing tetracycline from entering the cell.

## 4. Adverse Effects of Antibiotic Resistance

**Figure 3:**
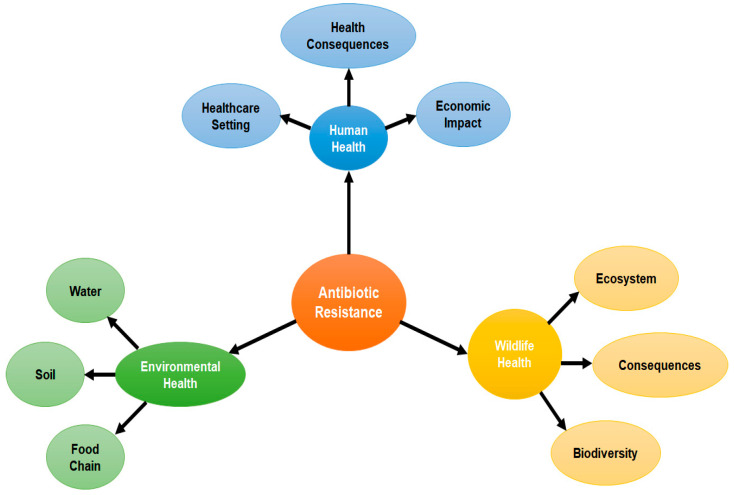
Impact and adverse effect of antibiotic resistance on health, wildlife, and environment.

Antibiotic resistance has been considered a global issue of public health nowadays, due to increasing resistance development among microbes against traditional antibiotics drugs, and it poses a need for new drugs. It is believed that the emergence of resistance to new antibiotics is probable and could limit the duration of these medications’ therapeutic efficacy. The World Health Organization (WHO) has declared the following so-called “ESKAPE” pathogens on the basis of their clinical significance and resistance levels: (E: *Enterococcus faecium*, S: *Staphylococcus aureus* or *Stenotrophomonas maltophilia*, K: *Klebsiella pneumoniae* or C: *Clostridioides difficile*, A: *Acinetobacter baumannii*, P: *Pseudomonas aeruginosa*, and E: *Enterobacteriaceae*). Resistance to antibiotics has an impact on every aspect of health, including the human, wildlife, and environmental sectors, affecting humanity as a whole (Figure 3). Antimicrobials are in fact frequently recommended in order to cure viral infections in both animals and humans, as well as to improve meat consumption in the food sector. 

Animal dung, industrial wastewater, treatment facilities, and organic wastes that are used in irrigation and fertilization of agricultural lands—all release a significant amount of antibiotics into the soil and water. Antimicrobial drugs that are released into the aquatic and soil ecosystem foster the growth of antifungal medication microorganisms and the emergence of antibiotic-resistant genes in the ecosystem [75]. There are multiple factors related to human activities (such as non-therapeutic and therapeutic use of antibiotics and the discarding of antibiotic formulations into the natural ecosystem) which induce resistance among natural microbial flora and which in turn affects human health when antibiotics fail to treat infectious diseases [76]. 

Antibiotic resistance has posed a major health concern, due to the growing number of bacterial infection among the human population as conventional antibiotics failed to control them. Antibiotic resistance has also emerged due to dose-related issues, a lack of accurate clinical disposal, and a lack of knowledge about the amount of antibiotics needed to treat disease [77]. As a result, distinct signs of pathogenic bacteria last until a sufficient amount is attained, at which point symptoms start to fade. Bacterial exposure to insufficient doses of these substances is thought to be the leading contributor to resistant pathogens, which may also be the root cause of bacterial development. In therapies, bacteria are typically faced with an excessively high concentration of just one or a few medications, while soil-borne microorganisms live in a diverse milieu and deal with a variety of stressors at the same time. These reactions most probably cancel out one another, favoring the evolution of antibiotic sensitivity over bacterial resistance [78]. Therefore, it is thought that human variables are to blame for the evolution of antibiotic resistance in clinical settings, which differs greatly from naturally found susceptibility. By simulating these natural habitats, it may be possible to understand how antibiotic resistance spreads among the microbial community [17]. 

Since the discovery of antibiotics, antibiotic resistance has challenged the scientific effort made by humanity to save them from infectious diseases. At the beginning of antibiotic discovery, resistance was not as challenging as it is in the 21st century, and the scientific research gaps and the need for new drug development were identified [79]. But recent developments in the profile of antibiotic resistance have forced the majority of drug companies to refocus their attempts on creating novel particles used in treating serious diseases [80]. This strategy offers more financial advantages that ensure their preservation in difficult economic times. As a result, the pathway for antibiotics may be blocked, leaving behind a few powerful compounds that may restrict the options for antibiotics used to treat particular diseases. The rise and spread of antibiotic-resistant organisms is a new danger to public health, necessitating worldwide action and a multifaceted strategy to address the fundamental difficulties involved in reducing drug resistance and to perfect the road forward [81].

## 5. Effects of Antibiotic Resistance on Drug Development: The Challenges

Modern clinical and scientific advancements in drug development are related to therapeutic success, and cope with different challenges in therapeutic fields. Many antibiotic advancements and developments are in the queue for coping with the public health emergency and issues of antibiotic resistance among the population [82]. Both biofilm and planktonic infections are the main targets of major therapeutic strategies imposed using antibiotics. The target of antibiotics is to stop the survival or growth of bacteria, including the synthesis of DNA and RNA or essential proteins, and the synthesis/maintenance of the cell wall. Several medications originated from substances that microbes have used to fight against one another for thousands of years [13]. Because bacteria have evolved the inherent ability to mutate and evade the damaging processes of many common antibiotics, the attacking instruments produced by microbes in this combat have triggered defense responses. Antibiotics used as a “last option” or in multiple or large dosages may be necessary to eradicate multidrug-resistant (MDR) microorganisms [83]. When microorganisms reside in biofilms, microbe resistance complicates the treatment challenge and frequently necessitates intense physical clearance of the biofilm via vigorous exfoliation, for instance, along with large doses of antibacterial therapy. The increased risks of adverse effects, antibiotic resistance and outcome failure have posed cost challenges for drug development and treatment [84].

According to reports from the Pew Trust (2017), the new drug development phases I to III will bring 39 new antibiotics, whose development is in progress. However, more research shows that the present antimicrobial pipeline is insufficiently strong to meet current and future clinical needs. Firstly, only 13 (33%) of the 39 antibiotics involved in research are likely to be developed into a medication that can be sold [85]. This is based on the treatment outcomes of progressing an antibiotic through various clinical phases [86]. Secondly, the majority of newly developed antibiotics lack the innovative modes of action or unique chemical compositions that attack well-validated targets required to considerably guarantee their efficacy against resistant pathogens [87]. A large number of the medicines involved in research are new formulations or mixtures of already existing substances. Finally, a number of these medications miss the most important infections that are resistant to antibiotics. According to the Pew Trust report, just 31% of medications being researched would indeed be effective against an ESKAPE infection, and 33% might be effective against that immediate-danger pathogen identified by the US Centers for Disease Control [24]. 

Significant capital firms have mostly abandoned the antibiotic market in favor of more lucrative therapeutic endeavors. Small and medium-sized businesses (SMEs) have made an effort to fill this gap, but they typically do not have the funds or capacity to embark on extensive and protracted research and development (R&D) [88]. As a result, the cheap fruit of antimicrobial research, such as chemical combination and renovations, has been picked. The costly and difficult challenge of identifying and creating genuinely innovative action mechanisms that are efficient in combating the most resilient diseases is now left [89]. 

Antibiotic resistance has posed a challenge to modern medicine, but has also enabled the world to evolve and has maintained the survival rates of children, youngsters, and the elderly. According to World Health Organization (2011) reports, antibiotic resistance has increased so much that if action is not taken against it, it could be incurable issue [90]. The disappearance of antibiotic drug development raises the threat of incurable infections. Antimicrobial Response is a new effort that has been started in response to the urgent need for action to avert this calamity. This action plan aims to develop and improve academic alliances, bring about the modification of expensive and time-consuming processes of regulation of antimicrobial drugs, and identify the economic growth of new antibiotics by trying to bring together populations that would need these substances with academic research, health practitioners, and the drug industry (cost of use vs. profit) [91]. A framework for these activities would be provided by a global partnership for the research and development of antimicrobial drugs [92].

The drug companies no longer view antibiotic research as a prudent financial venture. Antimicrobials are not nearly as valuable as medications that manage serious diseases like diabetes, mental disorders, asthma, or gastric reflux, since they are frequently therapeutic and only taken for brief periods of time [21]. The net present value (NPV) of a new medicine is really only around USD 50 million, according to a cost–benefit analysis conducted by the Department of Health Care in London, as opposed to nearly USD 1 billion for a medicine used to cure a neurodegenerative illness. Drug corporations prefer to make investments in chronic illness medications, since they are more valuable. The comparably low cost of antibiotics is another aspect that makes developing antibiotics unattractive from a business standpoint. In comparison to chemotherapeutic drugs [93], which can cost hundreds of thousands of dollars each course, modern antibiotics are typically priced at a minimum of USD 1000 to USD 3000 per course. Additionally, funders and the general populace consider antibiotics to be of low value because of their accessibility, simplicity of use, and usually inexpensive cost [94].

In contrast, experts in bacterial infections and microbiology have cautioned against overusing antibiotics. Due to their concern about fostering drug resistance, doctors frequently retain new antibiotics for use in the most severe instances rather than instantly giving them. Instead, they continue to use older antibiotics that have demonstrated equivalent efficacy. New medicines are therefore frequently used as “last-line” treatments to cure severe illnesses [95]. This technique results in a decreased return on investments and a decrease in the quantity of antimicrobial drugs. Resistance can ultimately develop when local agents are introduced, and this is almost certain. However, because microbial evolution is random, it is difficult to forecast when resistance may emerge [82]. Thus, when resistance to a new antibiotic arises, a company that has spent a lot of money on antibacterial drug research may find that their earnings are unexpectedly reduced. End-consumers of antibiotics have also been restrained by the Great Recession’s effects on the economy. The population now has access to inexpensive and usually effective medications, which is advantageous; on the other hand, many consumers anticipate that all antibiotics will be charged similarly, including novel treatments that target multi drug resistance (MDR) infections [96].

Due to extreme antibiotic resistance and multi drug resistance, large pharmaceutical companies have challenged and badly affected drug development, which needs millions of U.S. dollars of investment to design new antibiotics for the future. According to reports from the Infectious Diseases Society of America (IDSA) (2013), there are few antibiotics in phase 2 or 3 of development. The IDSA specifically cited the development of insufficiently few medicines that were active against developing extremely resistant Gram-negative pathogens such as *Enterobacteriaceae*, *Pseudomonas aeruginosa*, and *Acinetobacter baumannii*. Additionally, drug manufacturers are now more actively focused on creating medicines for Gram-negative infections than on methicillin-resistant *Staphylococcus aureus* (MRSA) [97]. The most probable reason for this disparity is that while MRSA is a significant issue globally, the market for treating Gram-negative bacteria is smaller and slightly more unexpected, due to the quick spread of resistance [98].

The process of discovering novel antibiotics and turning them into medicines is time- and money-consuming. A new drug must be developed, costing between USD 800 million and USD 1 billion, and it often takes more than ten years for it to reach the laboratory [99]. Examining alternatives to antibiotic therapy is a new strategy, given the urgency with which we must now combat antibiotic resistance. Another approach to combating antibiotic resistance is to prevent illnesses from occurring in the first place.

## 6. Latest Strategies against Drug-Resistant Microorganisms

The growing threat posed by drug-resistant bacteria is being intensively investigated by researchers and healthcare practitioners. The use of diverse methodologies, including bio-nanotechnology techniques, combinatorial medication approaches, and other ways, has recently advanced the treatment of antibiotic resistance [100,101,102]. The clinical effectiveness of the existing antibiotics and their recommended treatments are now seriously threatened by the epidemic of antimicrobial resistance [103,104]. The issue has several facets because it affects the social, financial, medicinal, and ecological sectors. To maintain the long-term accessibility of an effective therapy for infections, proper usage of currently available antibiotics is required in the absence of the advancement of new generations of antibiotic medicines [105]. Antibiotics may lose their effectiveness, which could result in a rise in mortality, the use of healthcare resources, and early mortality from infectious diseases that are already well-established and still pose a threat [106]. However, the increased utilization of antibiotics over the past 50 years has placed stress on bacteria that are more vulnerable to them, and may have helped resistant bacteria, a few of which are resistant to several antibiotics, to survive. Since sensitive bacteria may be more “fit” than bacterial resistance [107], it is anticipated that bacterial resistance may be replaced by susceptible bacteria if the overuse of antibiotics can be stopped [108]. 

Many research studies have been conducted on the origin, evolutionary aspect, and mechanisms of antibiotic resistance, but its effect on future drug development is poorly understood. Therefore, the current review will highlight the role of antibiotic resistance in drug design and the impacts of antibiotic resistance on drug development in the future. The most recent methods being tested to combat drug-resistant bacteria are listed in the Table 1. These tactics are a reflection of the dynamic and varied approaches being used to combat drug-resistant bacteria and guarantee successful treatments in the face of rising antimicrobial resistance.

(i) **New antibiotics:** The development of novel antibiotics targeting drug-resistant bacteria is a critical endeavor in the face of growing antimicrobial resistance. Researchers are actively seeking new treatment options to combat these resilient pathogens. One notable example is Teixobactin, a groundbreaking antibiotic discovered in 2015. Teixobactin effectively targets a wide range of drug-resistant Gram-positive bacteria [135]. Unlike some traditional antibiotics, Teixobactin’s mechanism of action, which disrupts bacterial cell walls, makes it less likely to trigger resistance [136]. Another innovative antibiotic, Lefamulin, is a pleuromutilin that received FDA approval in 2019 [137]. Lefamulin is used to treat community-acquired bacterial pneumonia, including infections caused by drug-resistant *Streptococcus pneumoniae*. It inhibits bacterial protein synthesis (binding to the peptidyl transferase center of the 50S bacterial ribosome, thus preventing the binding of transfer RNA for peptide transfer), providing an alternative treatment for cases where resistance to older antibiotics has emerged [138,139]. Zoliflodacin is being studied as a novel antibiotic for drug-resistant *Neisseria gonorrhoeae*, the bacteria responsible for gonorrhea [140]. This antibiotic, which falls into the spiroketone class, targets DNA replication, making it a promising option for combating a sexually transmitted infection that has developed resistance to multiple drugs [141]. Cefiderocol, a siderophore cephalosporin antibiotic, has been approved in several countries. It is highly effective against many drug-resistant Gram-negative bacteria, including carbapenem-resistant Enterobacteriaceae [142]. Cefiderocol works by disrupting bacterial cell walls and facilitating iron uptake, an innovative approach to addressing antibiotic resistance [143].
(ii) **Bacteriophages:** Bacteriophages (viruses that infect bacteria) produce lysins to break down bacterial cell walls, leading to bacterial death [144]. Engineered lysins have shown promise in targeting specific bacterial species or strains, including antibiotic-resistant ones. Lysins can be effective against a wide range of bacteria and can be developed relatively quickly. Lysins offer a potentially rapid response to bacterial infections, especially those caused by antibiotic-resistant strains [145]. However, there are challenges, such as lysin resistance and the potential for side effects due to the rapid release of bacterial toxins upon cell wall breakdown. Bacteriophages are viruses that infect and kill bacteria. They can be highly specific, targeting only certain bacterial strains. Phage therapy involves using specific phages to treat bacterial infections, including those caused by antibiotic-resistant bacteria. Phage therapy has been successfully used in some cases to treat antibiotic-resistant infections, particularly in regions where it has a history of use (e.g., Eastern Europe). However, challenges include finding the right phage for each infection, phage stability, regulatory hurdles, and the need for tailored treatments. Combining different approaches, such as antibiotics with antibodies, lysins, or phages, can enhance treatment effectiveness. This is because different strategies target different aspects of bacterial infection and resistance.
(iii) **Combination therapies:** The use of multiple antibiotics in combination is a promising strategy to enhance treatment efficacy and prevent the development of antibiotic resistance in bacteria. This approach involves administering two or more antibiotics with distinct mechanisms of action simultaneously. By targeting different aspects of bacterial physiology, combination therapy can reduce the selective pressure that drives resistance development. Two notable examples of combination therapies are Trimethoprim-Sulfamethoxazole (TMP-SMX) and Beta-lactam/beta-lactamase inhibitor combinations [115,116]. The TMP-SMX combination therapy pairs Trimethoprim, which inhibits bacterial dihydrofolate reductase, with Sulfamethoxazole (an inhibitor of dihydropteroate synthase). Together, these drugs disrupt two essential steps in the bacterial folate synthesis pathway. TMP-SMX is highly effective against a broad range of bacterial infections, including urinary tract infections, respiratory tract infections, and opportunistic infections in immunocompromised patients [146]. This combination is particularly valuable in treating methicillin-resistant Staphylococcus aureus (MRSA) and *Pneumocystis jirovecii pneumonia* [147]. The Beta-lactam/beta-lactamase inhibitor combinations are those in which a beta-lactam antibiotic (such as ampicillin, piperacillin, or cefotaxime) is paired with a beta-lactamase inhibitor (like sulbactam, clavulanic acid, or tazobactam). The inhibitor prevents the enzymatic degradation of the beta-lactam antibiotic, allowing it to remain effective against a broader spectrum of bacteria, including those with beta-lactamase enzymes. Examples of such combination therapies include ampicillin/sulbactam and piperacillin/tazobactam, which are used to combat various bacterial infections, especially those caused by beta-lactamase-producing organisms [148,149].
(iv) **Immune modulation:** Immune modulation is a strategy of boosting the immune response to enhance the clearance of bacterial infection and represents a critical strategy in addressing antibiotic resistance. The rise of drug-resistant bacteria has underscored the importance of fortifying the body’s natural defenses to complement antibiotic therapies and, in some cases, reduce the need for antibiotics. Several approaches have been explored to harness the immune system’s capabilities effectively. One of the strategies employed is the use of immunomodulatory compounds. These include cytokines such as interferons (e.g., interferon-gamma), interleukins, and colony-stimulating factors. Interferon-gamma, for instance, plays a pivotal role in activating macrophages, which are key components of the immune system. IFN-γ therapy has been utilized in treating infections like tuberculosis and non-tuberculous mycobacterial (NTM) infections [150]. By bolstering the immune response, these compounds help the body combat bacterial invaders more effectively. Vaccination is another proactive approach to stimulate the immune system. The development of vaccines targeting specific bacterial pathogens has yielded remarkable results. For instance, vaccines against drug-resistant strains of *Streptococcus pneumoniae* have significantly reduced the prevalence of antibiotic-resistant pneumonia [151]. Vaccination not only provides direct protection, but also curbs the spread of drug-resistant bacteria within communities. Immunostimulants, including granulocyte-colony stimulating factor (G-CSF), can be used to enhance white blood cell production [152]. This heightened immune response can assist the body in fighting off infections more effectively. While not a direct substitute for antibiotics, immunostimulants can be employed to reduce the reliance on antibiotics and support the immune system’s efforts [153].
(v) **Antibody therapies:** The development of monoclonal antibodies (mAbs) and antibody-derived molecules to target bacterial pathogens is an innovative approach in combating bacterial infections [154]. These engineered antibodies are designed to specifically recognize and neutralize bacterial targets, offering a precise and highly targeted method of treatment. Various strategies have been employed to harness the potential of these antibodies, with promising results. One notable example is the development of mAbs targeting *Clostridium difficile*, a bacterium responsible for antibiotic-associated diarrhea and colitis [155]. Bezlotoxumab, a monoclonal antibody, has been approved for the prevention of recurrent *C. difficile* infection. Bezlotoxumab works by binding to the *C. difficile* toxin B, preventing its harmful effects and reducing the risk of recurrent infections in high-risk patients [156]. This approach not only provides a targeted therapy, but also reduces the reliance on antibiotics, which can exacerbate *C. difficile* infections. Another groundbreaking development is the creation of mAbs targeting *Staphylococcus aureus*. In particular, the antibody-derived molecule called Altastaph is designed to bind to the bacteria’s surface protein, effectively neutralizing the pathogen. This approach is particularly relevant in combating methicillin-resistant *Staphylococcus aureus* (MRSA) infections, which are notorious for their resistance to multiple antibiotics [157]. By utilizing Altastaph and similar antibodies, researchers aim to enhance the immune system’s ability to recognize and clear MRSA infections. Beyond conventional mAbs, innovative antibody-derived molecules have been engineered. A notable example is that of lysibodies, which are designed to target a broad spectrum of bacteria by attacking conserved elements on the bacterial cell wall [158]. These molecules work similarly to mAbs, but have been engineered to recognize a wide range of bacterial species, reducing the need for highly specific antibodies for each pathogen. This approach offers the potential for a versatile and effective treatment strategy against a wide variety of bacterial infections. Researchers have also explored the use of antibody-conjugates, where antibodies are coupled with toxic agents to create a potent antimicrobial. One such example is the use of antibody–toxin conjugates targeting Pseudomonas aeruginosa [159]. By using antibodies to guide the toxin to the bacterial pathogen, this approach effectively destroys the bacteria and may represent a promising alternative to conventional antibiotics, especially in cases of multidrug-resistant strains.
(vi) **Nanotechnology:** Nanoparticles have emerged as a promising class of novel antimicrobials with diverse mechanisms of action and several advantages. One of their primary mechanisms is direct microbial inhibition, where their small size and high surface area allow them to physically disrupt microbial cell membranes. This can lead to cell lysis and the inactivation of microorganisms. Some nanoparticles, such as silver nanoparticles, release ions that interfere with microbial DNA replication and protein synthesis [160,161]. Additionally, certain nanoparticles can generate reactive oxygen species (ROS) when exposed to light. These ROS, including singlet oxygen and hydroxyl radicals, have potent antimicrobial properties which damage microbial components [162]. Nanoparticles can also inhibit biofilm formation, a crucial virulence factor in many pathogens. They achieve this by disrupting quorum sensing, preventing initial attachment, or destabilizing the biofilm matrix [163]. Moreover, nanoparticles can be designed for drug delivery, allowing the encapsulation and targeted release of antimicrobial agents directly at the infection site. This enhances drug stability and bioavailability, and minimizes systemic side effects, making them a valuable tool in antimicrobial therapy [164]. The advantages of nanoparticles as antimicrobials are manifold. They exhibit broad-spectrum activity against various microorganisms, including bacteria, fungi, and viruses. Their multiple mechanisms of action can slow down the development of antimicrobial resistance, a critical concern in contemporary medicine. Nanoparticles are also capable of enhancing the solubility of poorly soluble antimicrobial drugs, thereby increasing their therapeutic efficacy. Their ability to achieve targeted drug delivery minimizes damage to healthy tissues and maximizes the antimicrobial effect at the site of infection. Furthermore, nanoparticles offer stability to antimicrobial agents, protecting them from degradation and extending their shelf life. However, nanoparticles also come with certain limitations. Ensuring their biocompatibility with human cells and tissues is of paramount importance, particularly when considering in vivo applications. The cost of producing nanoparticles can be high, potentially limiting their widespread use, and regulatory challenges arise due to safety concerns. Achieving the specificity to target pathogens while sparing beneficial microorganisms remains a challenge. Additionally, the environmental impact of nanoparticles, especially their disposal into the environment, raises concerns regarding ecological consequences and the potential transfer of antibiotic resistance genes.

Metal–organic frameworks (MOFs) and their derived nanomaterials constitute a novel class of antimicrobial agents, showcasing exceptional diversity and promise in combating microbial threats [165]. MOFs are characterized by their unique crystalline porous structures, constructed from an amalgamation of metal ions or clusters with organic ligands, giving rise to more than 20,000 MOFs of varying topologies, morphologies, and dimensions, ranging from 0D nanoparticles to 3D hierarchical structures. MOFs have gained recognition for their remarkable advantages in antimicrobial applications. Their primary mode of antimicrobial action lies in the controlled liberation of metal ions from their intricate structures [166]. These liberated metal ions exert their effects by disrupting microbial cell membranes, interfering with intracellular processes, and generating reactive oxygen species (ROS), thereby inflicting oxidative stress and damaging microbial components. Moreover, MOFs can be meticulously engineered to encapsulate multiple drugs simultaneously, thus serving as a potent vehicle for combination therapies targeting bacterial infections through multiple mechanisms. This targeted drug delivery strategy enhances therapeutic efficacy, reduces off-target side effects, and mitigates the likelihood of resistance development. Additionally, it minimizes damage to host tissues and bolsters therapeutic impact at infection sites, demonstrating MOFs’ potential in alleviating antibiotic resistance by more efficiently delivering drugs to bacterial cells and lessening the selective pressure conducive to resistance development [167]. MOFs are versatile and can be tailored to exhibit broad-spectrum antimicrobial activity against an array of microorganisms, encompassing bacteria, fungi, and viruses. Their synthetic flexibility allows for the controlled and sustained release of various antimicrobial agents, ensuring their stability and maximizing bioavailability. Moreover, MOFs find potential applications in diverse fields, from wound dressings and coatings to implant materials aimed at averting infections. Nevertheless, MOFs do present certain limitations that warrant consideration. Ensuring MOF biocompatibility is a paramount concern, particularly when contemplating their use in in vivo settings, necessitating rigorous studies to assess potential toxicity to human cells and tissues. Furthermore, the elevated cost associated with MOF synthesis and the potential hurdles related to large-scale production could impede widespread utilization. Regulatory issues related to MOF application in antimicrobial contexts may also require attention. In addition, further research into the environmental impact of MOFs, encompassing their disposal, long-term stability, and potential ecological repercussions, is crucial [168].

Nanometer-sized carbon-based compounds called carbon quantum dots (CQDs) and carbon nanodots have special chemical, electrical, and optical characteristics [169,170]. They have been popular in a number of sectors, including bioimaging, drug delivery, and sensing applications [170]. Carbon quantum dots (CQDs) have emerged as a novel and promising class of antimicrobial agents with distinct mechanisms of action, notable advantages, and certain limitations. These nanoscale carbon-based materials exert their antimicrobial effects through multiple mechanisms. CQDs can directly interact with microbial cell membranes, disrupting their structural integrity and leading to the leakage of cellular components. They also generate reactive oxygen species (ROS), inducing oxidative stress that damages microbial biomolecules, such as proteins and nucleic acids. The ability of CQDs to target various components of microbial cells makes them effective against a wide range of microorganisms, including bacteria, fungi, and viruses [171]. The advantages of using CQDs as antimicrobials are multifaceted. Their nanoscale size allows for enhanced cellular uptake and bioavailability. Moreover, CQDs can be easily functionalized with specific antimicrobial agents, enhancing their targeted activity and reducing off-target effects [172]. Their biocompatibility is another significant advantage, making them suitable for various biomedical applications, such as wound dressings, coatings, and drug delivery systems. Additionally, CQDs exhibit stability and a long shelf life, further facilitating their practical use. However, there are certain limitations associated with CQDs as antimicrobials. Comprehensive studies are necessary to assess potential toxicity concerns when used in vivo. The synthesis of CQDs, while relatively straightforward, can still pose challenges for large-scale production and standardization. Regulatory aspects surrounding their use in medical applications need to be addressed. Additionally, their potential environmental impact and long-term stability, especially in disposal, require further investigation. In conclusion, CQDs represent a promising avenue in the development of novel antimicrobials, offering a range of advantages, but the challenges associated with their safety, production, and regulatory compliance must be addressed for their effective utilization in the fight against infectious diseases [173].

(vii) **Heterocyclic compounds:** Heterocyclic compounds, stemming from Thiazole, Imidazole, Thiazolidinone, Oxazole, Pyrrole, Pyridine, and Pyrimidine, constitute a highly diverse and essential class of organic chemicals. They have emerged as promising contenders in the quest for novel antimicrobial agents, thanks to their ability to employ a wide array of mechanisms that enhance their efficacy against various microbial threats. This family of compounds is increasingly in demand, due to its extensive applications in both the synthetic and biological domains. Notably, derivatives like Oxazole, Pyrrole, Pyranopyrimidine, and benzimidazole-based heterocyclic compounds exhibit distinct antibacterial properties against a broad spectrum of bacteria, encompassing both Gram-positive and Gram-negative strains [121]. Their primary mode of action revolves around disrupting essential biological processes within microorganisms, effectively inhibiting their growth and survival. This action includes the inhibition of vital enzymes, interference with nucleic acid synthesis, damage to cell membranes, and disruption of energy production pathways. Heterocyclic compounds offer the advantage of a well-understood synthetic chemistry, facilitating the creation of structurally diverse derivatives, thus enabling the optimization of their antimicrobial potential. Their versatility allows for the tailored development of compounds designed to target specific microorganisms or even strains that have developed resistance. However, it is essential to acknowledge the limitations associated with heterocyclic compounds, such as the risk of resistance development and potential toxicity concerns. Extensive research is needed to ensure their safety in clinical applications. Additionally, addressing regulatory considerations and overcoming challenges in large-scale production is vital to fully harness the potential of these compounds in the ongoing battle against infectious diseases [121].
(viii) **Antimicrobial peptides:** The exploration of naturally occurring peptides with antimicrobial properties has garnered significant attention in the search for alternative treatments against drug-resistant bacterial infections. These peptides, often referred to as antimicrobial peptides (AMPs), are naturally produced by various organisms, including humans, as part of their innate immune defense mechanisms [73]. AMPs exhibit a wide range of antimicrobial activities and have the potential to combat bacterial pathogens effectively. One remarkable example of an AMP is LL-37, which is a human host defense peptide found in various tissues and bodily fluids. LL-37 has demonstrated potent antimicrobial activity against a variety of bacteria, including drug-resistant strains [174]. It acts by disrupting bacterial cell membranes and interfering with intracellular processes. Researchers have been investigating the therapeutic potential of LL-37 in treating infections caused by multidrug-resistant bacteria, particularly in wound care and chronic skin conditions. Another group of naturally occurring peptides with antimicrobial properties is the defensins. Human defensins, such as human beta-defensins (hBDs), are small cationic peptides with broad-spectrum antimicrobial activity [175]. They can target both Gram-positive and Gram-negative bacteria, making them valuable candidates for developing novel treatments. For example, researchers are exploring the use of defensins in combination with traditional antibiotics to enhance their effectiveness against antibiotic-resistant strains. In nature, amphibians, such as frogs, produce a wide array of antimicrobial peptides as part of their defense mechanisms. Peptides like magainins and temporins have been isolated from the skin secretions of frogs and have demonstrated potent antibacterial activity [176]. These peptides function by disrupting bacterial membranes, ultimately leading to bacterial cell lysis. They have been the focus of research for their potential use in developing new antibiotics, especially for combating multidrug-resistant bacteria. Furthermore, peptides derived from various sources, including marine organisms, insects, and plants, have shown promise as antimicrobial agents. For instance, researchers have investigated the use of peptides from marine sponges and algae to combat bacterial infections. Additionally, antimicrobial peptides from bee venom, like melittin, have displayed activity against a range of pathogens [177].
(ix) **CRISPR-Cas system:** The application of CRISPR gene-editing technology to target and eliminate drug-resistant bacteria represents a groundbreaking approach in the fight against antibiotic resistance. CRISPR, which stands for Clustered Regularly Interspaced Short Palindromic Repeats, is a revolutionary tool that allows precise modification of an organism’s DNA, including bacteria. By leveraging CRISPR, researchers can engineer specific genetic changes to disrupt drug resistance mechanisms in bacterial pathogens. One remarkable example of using CRISPR technology to combat antibiotic resistance is the development of phage therapy [178]. Phages are viruses that can infect and kill bacteria. Researchers have employed CRISPR to modify phages so that they target antibiotic-resistant bacterial strains. By introducing CRISPR-modified phages into the bacterial population, scientists can selectively eliminate resistant bacteria, restoring susceptibility to antibiotics. This approach offers a promising strategy for overcoming drug resistance and enhancing the effectiveness of conventional antibiotics. Another example involves the modification of a bacteria’s own genes to reverse antibiotic resistance. Researchers have used CRISPR technology to target and disrupt the genes responsible for antibiotic resistance in bacterial strains [125]. By doing so, they can restore the susceptibility of these bacteria to antibiotics that were previously ineffective. This approach may be particularly useful in clinical settings, where antibiotic-resistant infections can be life-threatening. Moreover, CRISPR-based gene editing has enabled the development of novel antimicrobial compounds. Scientists have used this technology to design synthetic peptides or proteins that specifically target and inhibit drug-resistant bacterial mechanisms. These customized molecules can interfere with essential bacterial processes, ultimately rendering antibiotic-resistant strains vulnerable to traditional antibiotics. This innovative approach opens up new avenues for developing antimicrobial agents. CRISPR technology’s versatility allows for the customization of strategies to combat antibiotic resistance in a highly targeted manner. Researchers can design CRISPR sequences to recognize and disrupt various resistance mechanisms, such as efflux pumps or antibiotic-modifying enzymes [179]. As a result, drug-resistant bacteria become more susceptible to existing antibiotics. The utilization of CRISPR gene-editing technology to target and eliminate drug-resistant bacteria has the potential to transform the field of antimicrobial research and clinical practice. However, there are challenges to overcome, including the need for safe and efficient delivery methods for CRISPR components. Ensuring the specificity and accuracy of CRISPR targeting is crucial to avoid unintended consequences. Nonetheless, the development and refinement of CRISPR-based strategies provide hope for combating antibiotic resistance and extending the effectiveness of existing antibiotics.
(x) **One Health approach:** A holistic approach integrating human health, animal health, and the environment is crucial in addressing antibiotic resistance. Recognizing the interconnectedness of these elements is vital to combat this global challenge. In such an approach, human health and antibiotic use are considered in conjunction with animal health and environmental factors [180]. One example of this approach is the One Health initiative, which emphasizes the interplay between humans, animals, and the environment. It encourages collaborative efforts between healthcare professionals, veterinarians, environmental experts, and policymakers to mitigate antibiotic resistance. In the context of human health, this approach involves promoting responsible antibiotic use in healthcare settings. This includes optimizing prescription practices to reduce overuse and misuse of antibiotics [181]. In animal health, it pertains to the judicious use of antibiotics in veterinary medicine and agriculture. Implementing stricter regulations on antibiotic use in livestock and aquaculture helps minimize the risk of antibiotic-resistant bacteria entering the food chain. Furthermore, the environmental aspect involves understanding how antibiotic residues and resistant bacteria can impact ecosystems. The presence of antibiotics in the environment can promote the selection of resistant strains. This holistic approach necessitates proper disposal of pharmaceutical waste and controlling the release of antibiotics into water sources [182].
(xi) **Drug repurposing:** Investigating existing drugs for potential activity against drug-resistant microorganisms is a promising strategy in the battle against antibiotic resistance [4,183]. This approach involves reevaluating established medications to identify whether they possess antimicrobial properties, even if that was not their primary purpose. This repurposing of drugs can provide effective treatments for drug-resistant infections. An example of this is the use of statins, commonly prescribed for managing cholesterol levels, which have shown potential antibacterial properties [184]. Statins can disrupt bacterial membranes and potentially enhance the action of traditional antibiotics. Moreover, other drugs used for non-antibiotic purposes have demonstrated antimicrobial activity. For instance, antipsychotic agents like chlorpromazine and thioridazine, which treat mental health conditions, have been explored for their ability to inhibit bacterial efflux pumps and increase the susceptibility of bacteria to antibiotics [185]. This approach maximizes the utility of existing drugs and can be particularly valuable when combating multidrug-resistant pathogens. A key advantage of investigating existing drugs is the wealth of safety and efficacy data available. These drugs have already undergone extensive testing in human patients, which can expedite their repurposing for antimicrobial applications. By identifying non-traditional antimicrobial agents among existing drugs, the antibiotic arsenal can be enhanced and new treatments for drug-resistant microorganisms can be offered.
(xii) **Surveillance and diagnostics:** Improved monitoring and diagnostic tools are crucial for the early detection and tracking of resistance in microorganisms, ensuring more effective responses to the threat of antibiotic resistance. One example of this is the development and utilization of rapid molecular diagnostics, such as polymerase chain reaction (PCR) tests, which can quickly identify the presence of antibiotic resistance genes in bacterial samples [186]. This technology enables healthcare professionals to make informed decisions regarding the choice of antibiotics and treatment regimens, improving patient outcomes and helping contain resistance. Furthermore, advances in metagenomics, particularly next-generation sequencing techniques, have enabled researchers to monitor the entire microbial community within a given environment. It is especially valuable in tracking the emergence of new resistance mechanisms and identifying areas where antibiotic-resistant bacteria are prevalent [186]. In addition, digital health tools and telemedicine have been harnessed to enhance surveillance and monitoring. These technologies facilitate the real-time reporting of antibiotic-resistant infections, aiding healthcare authorities in responding promptly to outbreaks and implementing containment measures [187]. Efforts to improve monitoring and diagnostics also extend to the development of biosensors and microfluidic devices capable of detecting antibiotic resistance at the point of care. For instance, researchers have designed microchip-based devices that can rapidly identify drug-resistant bacteria by analyzing their responses to antibiotics [188]. These tools offer the potential for on-site diagnostics and tailored treatment decisions.

## 7. Safety, Efficacy and Commercial Viability of Common Alternative Therapies

The safety, efficacy, and commercial viability of common alternative therapies in comparison to antibiotics are critical aspects to consider when combating bacterial antibiotic resistance. Overall, it depends on the specific therapy and the targeted bacterial infections. One such alternative therapy is phage therapy, which involves using bacteriophages, viruses that infect and kill bacteria. Phage therapy offers several advantages, including high specificity for bacterial strains, potentially lower toxicity compared to antibiotics, and the ability to adapt to evolving bacterial resistance. However, it faces challenges related to regulatory approval and commercial production scalability, which can impact its widespread use. Another alternative therapy is the use of probiotics, which are beneficial bacteria that can help restore a healthy microbial balance in the body. Probiotics have the advantage of being generally safe and can prevent infections by competing with pathogenic bacteria. However, their efficacy can vary depending on the strain and the condition they are used to treat. The commercial viability of probiotics is well-established, with a wide range of products available to consumers. Essential oils and plant extracts are natural compounds that have shown promise in combating antibiotic-resistant bacteria. These alternatives have the advantage of being generally safe and readily available. They can disrupt bacterial cell membranes and inhibit essential metabolic processes. However, challenges exist in standardizing their composition, which can affect their efficacy and commercial viability. Nanoparticles, such as silver nanoparticles, have demonstrated antibacterial properties. They offer the advantage of versatility in design and the potential for controlled drug delivery. However, their safety, especially in long-term use, needs further evaluation, and the commercial production of high-quality nanoparticles can be costly. Antimicrobial peptides (AMPs) are naturally occurring molecules with potent antibacterial properties. They have the advantage of being highly selective for bacterial targets and with a lower potential for resistance development. Commercialization of AMP-based therapies is still in the early stages, with challenges related to large-scale production and cost-effectiveness. Monoclonal antibodies and antibody-derived molecules are emerging as alternative therapies to combat bacterial antibiotic resistance. These highly specific molecules can be tailored to target bacterial pathogens effectively. They have demonstrated safety and efficacy, particularly in cases where bacterial surface proteins are targeted to prevent infection. Examples include antibodies against *Clostridium difficile* and *Staphylococcus aureus*. However, the commercial viability of monoclonal antibodies depends on factors like research, manufacturing costs, regulatory approval, and accessibility. Furthermore, the safety, efficacy, and commercial viability of all these alternative strategies are summarized in Table 2. 

## 8. Limitations and Challenges in the Development of New Antibiotics

The antimicrobial pipeline, particularly in the context of antibiotics, faced several limitations that made it insufficient to meet clinical needs. These limitations were primarily driven by challenges and bottlenecks in the antibiotic development process, such as [195]:

(i) Limited Novel Antibiotic Discovery:

Discovering new antibiotics with novel mechanisms of action is becoming increasingly difficult. The low-hanging fruit in terms of antibiotic discovery has been largely explored, and finding compounds that can effectively target resistant bacteria is a significant challenge [196,197]. The lack of novel antibiotics means that there are fewer options available to combat antibiotic-resistant infections. This limitation is especially problematic when dealing with multidrug-resistant bacteria.

(ii) Antibiotic Resistance Development:

One of the most significant challenges is the rise of antibiotic-resistant bacteria. Over time, many bacteria have developed resistance to existing antibiotics due to the overuse and misuse of these drugs [198]. This has rendered some antibiotics ineffective, limiting the treatment options for bacterial infections. Bacteria have evolved and developed resistance mechanisms against many existing antibiotics. This makes previously effective treatments ineffective and increases the risk of untreatable infections. The growing prevalence of antibiotic-resistant bacteria reduces the effectiveness of current antibiotics, leading to a diminishing arsenal of effective treatment options [199].

(iii) Limited Spectrum of Activity:

Many antibiotics have a specific spectrum of activity, meaning they are effective against only certain types of bacteria. This limitation becomes problematic when treating infections caused by bacteria that are not susceptible to the available antibiotics.

(iv) Complex Clinical Trials:

Conducting clinical trials for antibiotics presents unique challenges, such as recruiting patients with specific infections, determining appropriate endpoints, and accounting for the rapid progression of certain infections [200]. These challenges can lead to delays in clinical trial completion, slowing down the overall development process.

(v) Need for Combination Therapies:

Some infections require combination therapy with multiple antibiotics to achieve optimal effectiveness and prevent resistance [201]. Developing effective combinations and determining appropriate dosages is complex. Developing and testing combination therapies adds to the challenges and costs of antibiotic development.

(vi) Stewardship and Responsible Use:

Promoting responsible antibiotic use is critical to slowing the development of resistance. However, striking a balance between making new antibiotics available and preserving their efficacy through responsible use is a challenge [202]. Overuse and misuse of antibiotics contribute to resistance development, making it difficult to extend the lifespan of new antibiotics [203].

## 9. Hurdles and Challenges in Regulatory Process for New Antibiotics

Numerous obstacles and challenges within the regulatory landscape impede the advancement of new antibiotics. These hurdles can lead to development delays, hinder investment, and curtail the availability of effective treatments [204]. Regulatory agencies exercise caution in approving new antibiotics due to their concern over the rapid emergence of resistance. Consequently, this cautious approach results in stricter demands for proving the efficacy and safety of these novel antibiotics. Traditional clinical trial designs pose another challenge, particularly when rapid patient enrollment and treatment are pivotal. Moreover, conventional endpoints may fall short in capturing the full impact of antibiotic therapies on patient well-being. Furthermore, enlisting patients with specific resistant infections for clinical trials proves arduous, given the rarity of such infections and the need for meticulous patient selection. The variability in bacterial resistance patterns further complicates matters, potentially altering during the course of clinical trials and thereby influencing the interpretation of trial outcomes.

A significant obstacle faced by pharmaceutical companies is the lack of alignment between economic incentives for antibiotic development and the associated costs and risks. Antibiotics’ low profit margins and brief treatment periods diminish their appeal to these companies. Additionally, bringing new antibiotics into the market presents difficulties due to apprehensions about resistance, intricacies in conducting clinical trials, and challenges related to reimbursement.

The absence of transparent and efficient regulatory pathways tailored specifically for antibiotics can impede the approval process and extend the time needed for development. The lack of comprehensive regulatory guidance for certain facets of antibiotic development, such as combining therapies or devising adaptable trial designs, has the potential to sow uncertainty and obstruct progress. While it is crucial to monitor the safety and efficacy of antibiotics post approval, establishing effective mechanisms for post-approval surveillance and data collection can prove challenging. Discrepancies in regulatory standards and requisites across different countries can introduce additional complications in the global pursuit of antibiotic development and approval. Some regulatory bodies and assessors might lack experience in evaluating antibiotics with novel mechanisms of action or those engineered to target specific resistance mechanisms. 

## 10. Modification in Regulatory Process to Facilitate Antibiotic Development

To facilitate antibiotic development and address the challenges posed by antibiotic resistance, several specific changes or improvements in the regulatory process are necessary. This may include the following:

(i) Adaptive Pathways and Flexible Trial Designs: The regulatory process should allow adaptive trial designs that can be adjusted based on emerging data. This flexibility can speed up the development process and accommodate the evolving nature of bacterial resistance.
(ii) Streamlined Approval Process: Establishment of a streamlined regulatory pathway specifically for antibiotics that addresses their unique challenges. This could involve expedited reviews, accelerated approvals, or priority designations for antibiotics targeting urgent or unmet medical needs.
(iii) Tailored Clinical Trial Endpoints: Development of clinically meaningful endpoints for antibiotic trials that reflect their unique mechanism of action and intended use. These endpoints should consider both short-term efficacy and long-term impact on antibiotic resistance.
(iv) Guidance on Combination Therapies: The regulatory process should provide clear guidance on developing and testing combination therapies involving antibiotics, antibodies, probiotics, or other approaches. This guidance should address dosing, interactions, and potential synergies.
(v) Biomarker Development: Investment in research to identify predictive biomarkers that can indicate early in clinical trials whether an antibiotic is effective. Biomarkers can help streamline development and reduce trial durations.
(vi) Economic Incentives: Offering economic incentives such as extended market exclusivity or market entry rewards for antibiotics that target high-priority pathogens or mechanisms of resistance [205].
(vii) Adaptive Licensing: Implementation of adaptive licensing strategies that allow earlier access to antibiotics based on initial safety and efficacy data, with continued monitoring and data collection post approval.
(viii) Collaborative Approaches: Encouragement of collaboration between regulatory agencies, industry, academia, and public health organizations to share data, insights, and best practices for antibiotic development and regulatory evaluation.
(ix) Real-World Evidence: Incorporation of real-world evidence, such as data from patient registries and observational studies, to supplement traditional clinical trial data and support post-approval evaluations.
(x) Global Harmonization: Working towards global harmonization of regulatory standards for antibiotic development. This can reduce duplication of efforts and create a more efficient pathway for international approvals.
(xi) Antibiotic Stewardship Education: Incorporation of antibiotic stewardship education and responsible-use recommendations into regulatory processes to ensure that new antibiotics are used appropriately to slow the development of resistance.

These changes and improvements aim to strike a balance between fostering innovation, ensuring patient safety, and addressing the urgent need for effective antibiotics in the face of growing antibiotic resistance. Collaborative efforts between regulatory agencies, industry stakeholders, healthcare providers, and researchers will be crucial in implementing these modifications effectively.

## 11. One Health Approach

The intricate interplay between human, animal, and environmental health exerts a profound influence on the emergence and dissemination of antibiotic resistance. This complex web of interactions has far-reaching consequences for public health and necessitates a multifaceted approach to comprehensively address the challenges posed by antibiotic resistance [180]. The One Health approach underscores the dynamic interactions between humans, animals, and the environment and advocates for collaborative efforts among healthcare professionals, veterinarians, environmental experts, and policymakers to mitigate antibiotic resistance [181]. 

Human health is significantly impacted by antibiotic resistance due to the misapplication and excessive use of antibiotics in clinical settings. It contributes to the selection of antibiotic-resistant pathogens, leading to infections that are challenging to treat. Consequently, it results in prolonged illnesses, heightened healthcare costs, and increased mortality rates. Furthermore, individuals may unknowingly harbor antibiotic-resistant bacteria, serving as potential reservoirs for further transmission. On the animal health front, the One Health approach advocates for responsible antibiotic use in veterinary medicine. It includes judicious antibiotic use in the treatment and prevention of diseases in animals, from livestock to pets. Disease prevention strategies and heightened surveillance of antibiotic-resistant pathogens in animals are also key components [182]. The third key component of the One Health approach, i.e., environmental health, plays a crucial role in the spread of antibiotic resistance. Antibiotics and antibiotic-resistant bacteria can enter the environment through various means, including agricultural runoff and wastewater. A One Health approach calls for proper disposal of expired antibiotics, the monitoring of antibiotic levels in water sources, and for measures to reduce environmental contamination. 

Surveillance and data sharing are vital aspects of a One Health strategy. Effective surveillance systems that track antibiotic-resistant pathogens in humans, animals, and the environment are essential. Collaboration and data sharing among human and animal health agencies, environmental agencies, and research institutions are encouraged to build a comprehensive understanding of antibiotic resistance trends. Research and innovation are key drivers in the fight against antibiotic resistance. A One Health approach promotes research into novel antibiotics, alternative therapies, and vaccine development to combat antibiotic-resistant infections. By investing in research and development, new treatment options can be explored, reducing reliance on existing antibiotics. Regulatory and policy frameworks are integral to a One Health strategy. Policymakers are responsible for enforcing regulations that govern antibiotic use in human, animal, and environmental domains. Developing and implementing policies that encourage responsible antibiotic use and limit misuse and overuse are central to the approach. International collaboration is a critical component of a One Health approach. Antibiotic resistance is a global issue that transcends borders. By fostering international collaboration, countries can work together to address the spread of antibiotic-resistant pathogens and the movement of antimicrobial resistance genes. Education and awareness campaigns are also essential in promoting the One Health approach. Public awareness efforts aim to inform people about the dangers of antibiotic resistance, the importance of completing prescribed antibiotic courses, and the broader implications of overuse. Education should target healthcare professionals, veterinarians, farmers, and the general public.

Several examples and case studies demonstrate the effective implementation of the One Health approach to mitigate antibiotic resistance:

(i) The Netherlands’ Success in Controlling MRSA: The Netherlands adopted a One Health strategy to tackle methicillin-resistant *Staphylococcus aureus* (MRSA) in livestock and subsequently in humans [206]. They introduced strict regulations on antibiotic use in agriculture and implemented effective surveillance programs. This led to a significant reduction in MRSA prevalence in both animals and humans.
(ii) The Danish Integrated Antimicrobial Resistance Monitoring and Research Program: Denmark has been proactive in implementing a One Health approach to address antibiotic resistance [207]. Their Integrated Antimicrobial Resistance Monitoring and Research Program (DANMAP) tracks antibiotic usage and resistance in humans, animals, and food. As a result, Denmark has seen a decrease in the use of antibiotics in animal farming and a subsequent reduction in resistance.
(iii) The Case of Nipah Virus in Bangladesh: The emergence of the Nipah virus in Bangladesh was tackled through a One Health approach [208]. Health authorities coordinated with veterinary services, ecologists, and other stakeholders to investigate the source and transmission routes of the virus. This collaborative effort allowed for a better understanding of the virus, leading to more targeted public health interventions.
(iv) The Swedish Strategy Against Antibiotic Resistance: Sweden implemented a One Health strategy that involves close collaboration between human and veterinary medicine [209]. This approach has resulted in low antibiotic consumption in both healthcare and agriculture, contributing to low levels of antibiotic resistance.
(v) Tackling Zoonotic Diseases in Africa: Various African countries have embraced the One Health approach to combat zoonotic diseases [210]. For instance, Kenya established the Zoonotic Disease Unit (ZDU), which works to control diseases that spread between animals and humans. ZDU promotes collaborative efforts involving healthcare workers, veterinarians, and environmental experts to address diseases like rabies and anthrax.
(vi) The Work of the FAO, WHO, and WOAH: The Food and Agriculture Organization (FAO), World Health Organization (WHO), and World Organization for Animal Health (WOAH) have been collaborating to address antibiotic resistance at a global level [211]. They work on guidelines for responsible antibiotic use in agriculture and the surveillance of resistance in both animals and humans.
(vii) Global Initiatives on Tuberculosis: Tuberculosis (TB) is an example where the One Health approach is critical. Organizations like the Stop TB Partnership and the World Health Organization work with veterinarians to tackle TB in cattle, as it can also infect humans [212,213]. By addressing the disease in both humans and animals, they aim to reduce the overall burden of TB.

Integrated approaches, such as the One Health Approach, have demonstrated notable achievements in the battle against antibiotic resistance. One of the most significant triumphs of the One Health Approach has been the substantial reduction in antibiotic usage, particularly within animal agriculture. Nations such as Denmark and the Netherlands have effectively curtailed the application of antibiotics in animal farming through their holistic approaches, resulting in diminished resistance rates. Integrated strategies have proven highly efficacious in diminishing the prevalence of antibiotic-resistant strains. These successful programs have translated into a tangible decrease in infections caused by resistant bacteria, both in human healthcare and veterinary settings. These integrated approaches have significantly enhanced surveillance and facilitated comprehensive data sharing. Collaborating across different sectors has resulted in a more profound understanding of antibiotic resistance patterns, thus providing the basis for targeted interventions and policy modifications. Integrated surveillance systems have played a pivotal role in the early detection of emerging health threats. This holds particular importance in the context of zoonotic diseases, where the identification and mitigation of issues at their source can pre-empt outbreaks in human populations. Several nations and international organizations have adeptly formulated policies and regulations designed to restrict antibiotic use in animal agriculture. These policies are rooted in scientific evidence and engage stakeholders from a multitude of sectors. This collaborative and evidence-based approach has been instrumental in the development of guidelines that foster responsible antibiotic use and reduce the emergence of resistance in both animals and humans.

The One Health Approach, while highly effective, is not without its share of challenges, encompassing intricate coordination, limited resources, regulatory hurdles, a lack of global consensus, environmental complexities, behavioral shifts, and the imperative for worldwide collaboration. Implementing the One Health approach demands intricate coordination and collaboration across multiple sectors, a task that can be inherently complex. Diverse agencies and stakeholders often harbor varying priorities and interests, making cohesive management a formidable undertaking. The integrated nature of the One Health approach can be resource-intensive. Gathering, analyzing, and sharing data spanning various sectors, in addition to implementing policies and interventions, necessitates substantial funding and infrastructure. Enforcing policies related to antibiotic usage in agriculture can be a formidable challenge. Resistance may emerge from certain quarters, and monitoring and regulating practices within the animal farming industry can pose difficulties. Attaining global consensus on the matter of antibiotic resistance and its interconnectedness with human, animal, and environmental health remains an ongoing challenge. Not all nations have embraced integrated strategies, potentially resulting in issues related to resistance transcending national borders. The environmental dimension of the One Health approach is highly intricate. The consequences of antimicrobial residues in ecosystems and water sources have not been comprehensively elucidated. Furthermore, mitigating resistance within the environment poses particular challenges. Influencing behavioral changes in healthcare practices, such as the reduction in antibiotic prescriptions, and in agricultural practices, like curbing antibiotic use in animals, can be a gradual and demanding process, necessitating educational efforts and shifts in societal norms. International cooperation stands as a vital component of effectively combating antibiotic resistance. However, it comes with its set of challenges, including the assurance that all nations adhere to best practices and make a steadfast commitment to address this pressing issue.

## 12. Economic Implications of Antibiotic Resistance and Its Impact on Drug Development

The economic implications of antibiotic resistance are substantial and multifaceted, with wide-ranging effects on healthcare costs, drug development, and the global economy. These implications pose significant challenges to healthcare systems, pharmaceutical industries, and society as a whole. The World Bank models estimate that under a low burden of antimicrobial resistance, health costs could increase to USD 330 billion; under a high-burden scenario, this increase could be USD 1.2 trillion [214]. Here, we delve into the economic aspects of antibiotic resistance:

(i) Increased Healthcare Costs: One of the most immediate economic consequences of antibiotic resistance is the rise in healthcare costs [215]. Resistant infections often require more extended hospital stays, complex treatments, and costly second-line antibiotics. Further, the increased financial burden on healthcare systems can limit resources available for other critical health services.
(ii) Lost Productivity: Antibiotic-resistant infections can lead to extended sick leave, decreased productivity, and even disability or death, resulting in economic losses [216]. When individuals are unable to work or need prolonged medical care due to antibiotic-resistant infections, it affects not only their personal income but also overall workforce productivity.
(iii) Antibiotic Research and Development Costs: Developing new antibiotics is a costly and time-consuming process. The pharmaceutical industry faces financial disincentives to invest in antibiotic research and development because the market for these drugs is limited [205]. Unlike chronic conditions where patients may take medications for an extended period, antibiotics are typically taken for short durations. Therefore, the return on investment for new antibiotics is often lower than for drugs used to treat chronic diseases.
(iv) Market Dynamics: The pharmaceutical market dynamics play a significant role in the economic implications of antibiotic resistance [217]. As new antibiotics face challenges in reaching the market, existing antibiotics may experience price increases. Drug shortages, which are often linked to production and distribution issues, can lead to higher prices for antibiotics, impacting both healthcare facilities and patients.
(v) Global Trade and Food Production: Antibiotic resistance can have indirect economic consequences through its impact on global trade and food production [218]. In agriculture, the use of antibiotics in animal husbandry can lead to the development of resistant bacteria and contribute to the spread of antibiotic resistance. This can disrupt food supplies, increase production costs, and potentially affect food prices, impacting both the agricultural and food industries.
(vi) Tourism and Travel: Resistant infections can discourage tourism and travel to regions with higher prevalence rates [219]. Countries with reputations for antibiotic-resistant healthcare-associated infections may see a decline in medical tourism and tourism in general. This can have a direct impact on local economies that rely on tourism as a significant revenue source.
(vii) Innovative Treatments: The emergence of antibiotic resistance can also drive innovation in medical treatments. Researchers and healthcare professionals are exploring alternative treatment options, such as phage therapy and monoclonal antibodies, which may have economic implications in terms of development costs and market competition [220].
(viii) Global Economic Impact: Antibiotic resistance is a global issue that can have international economic repercussions. The spread of resistant strains across borders can impact trade, tourism, and international relations. Collaborative efforts to address the problem and share best practices can mitigate these economic consequences [182].

## 13. Healthcare Costs Associated with Antibiotic-Resistant Infections, and the Potential Return on Investment for Pharmaceutical Companies

Healthcare costs associated with antibiotic resistance have become a substantial burden on healthcare systems worldwide. The financial implications of treating patients with resistant infections are multifaceted and extend across different aspects of healthcare delivery. Longer hospital stays, specialized treatments, and increased diagnostic expenses contribute to the rising healthcare costs [221]. Patients with resistant infections often require extended care, as the treatment becomes more challenging due to limited antibiotic options. This leads to an increase in hospitalization costs, a heavier demand for resources, and the need for additional staff to manage the complex cases. Furthermore, advanced laboratory testing and diagnostics are crucial for accurate diagnosis and monitoring of resistant infections, which adds to the financial burden. Identifying the resistance profile of the infecting pathogen is essential for guiding treatment decisions. The costs associated with laboratory tests and diagnostic procedures can be substantial. Infection control measures are another significant contributor to healthcare costs. To prevent the spread of resistant infections within healthcare facilities, stringent infection control practices are necessary. These practices include isolating infected patients, implementing thorough hygiene protocols, and dedicating additional resources to maintaining a clean and safe environment [198]. The financial investment required to establish and maintain these measures places additional pressure on healthcare budgets. The allocation of resources, such as personnel, bed capacity, and equipment, also needs to be adjusted to accommodate patients with resistant infections. Overall, the economic implications of antibiotic resistance are substantial and extend far beyond the direct costs of antibiotic treatments. For pharmaceutical companies, the development of new antibiotics presents unique challenges and economic considerations. The limited market size for antibiotics, combined with high development costs and the ongoing emergence of resistance, has made antibiotic research and development less attractive in comparison to drugs for chronic conditions. Antibiotics are often prescribed for relatively short durations, which restricts their revenue potential. The rigorous and costly clinical trial process, coupled with the uncertainty of a drug’s success, further complicates the return on investment. Many potential antibiotic candidates do not progress to market approval. Pharmaceutical companies also face competition with older, generic antibiotics. These generic alternatives are typically available at lower prices, making it challenging for companies to recoup development costs and generate significant profits from new antibiotics [222]. The relatively small market for antibiotics means that the return on investment is less predictable and attractive to pharmaceutical companies. To mitigate these challenges and incentivize antibiotic development, various policy measures have been proposed. These include financial incentives, extended market exclusivity for new antibiotics, and public–private partnerships. Governments and international organizations play a pivotal role in fostering an environment that encourages pharmaceutical companies to invest in antibiotic research and development.

## 14. Withdrawal of Major Capital Firms in Antibiotic Research: Reasons and Strategies to Re-Engage Them

Major capital firms have shifted their focus away from antibiotic research, due to a multitude of reasons. These encompass factors such as low profit margins, steep development costs, regulatory complexities, challenges in market entry, and a tendency to prioritize alternative therapies [223]. Antibiotics are typically priced lower than other pharmaceuticals due to their shorter treatment durations. Consequently, this leads to diminished profit potential for pharmaceutical companies, rendering antibiotic development financially less appealing. The process of developing antibiotics incurs substantial research and development expenses, including clinical trials, regulatory approval, and manufacturing. However, these antibiotics are often retailed at comparatively modest prices in relation to other pharmaceuticals, mainly because of their shorter therapeutic courses. This combination of elevated development costs and constrained profit margins has created a disincentive for pharmaceutical companies to invest extensively in antibiotic research and development [223]. 

Introducing new antibiotics to the market faces considerable challenges driven by concerns surrounding resistance, intricacies of clinical trial design, and complexities tied to reimbursement. This results in new antibiotics encountering obstacles when attempting to gain a foothold in the market. These hurdles impede their commercial viability and may discourage companies from committing resources to their advancement. Adding to this landscape, regulatory agencies impose stringent requirements for demonstrating the safety and efficacy of new antibiotics, given the looming threat of antibiotic resistance [224]. Consequently, the approval process for these antibiotics can be prolonged and more demanding.

The stringent regulatory environment can, unfortunately, create a deterrent for companies seeking to pursue antibiotic development, consequently decelerating the introduction of innovative treatments. Consequently, many major capital firms prioritize investments in domains where they foresee greater potential for substantial financial returns. As a result, their investments often lean towards chronic disease treatments and therapies that offer more protracted revenue streams. This trend underscores the importance of addressing the economic misalignment in antibiotic research and development, fostering an environment that recognizes the urgency of combating antibiotic resistance while acknowledging the financial realities faced by pharmaceutical companies.

To incentivize the participation of major capital firms in antibiotic research, a range of strategies and motivating factors can be explored. Governments and public health entities hold the potential to extend financial incentives to companies that successfully introduce novel antibiotics to the market. This could encompass providing subsidies, grants, or even secured purchases to ensure a reasonable return on their investment. Additionally, there is the option for governmental and public health bodies to offer substantial monetary rewards as an incentive for the creation of critically needed antibiotics that fulfill specific criteria, such as targeting specific types of antibiotic-resistant bacteria. 

Incorporating mechanisms to extend the period of market exclusivity, during which generic versions are restricted, could amplify the allure of antibiotic development for capital firms. Furthermore, fostering collaborations between governmental bodies, academic institutions, and pharmaceutical corporations presents an avenue to share the burdens and uncertainties intrinsic to antibiotic research and development. This collective approach could heighten the appeal for capital firms to invest. 

Extending the shield of patent protection for antibiotics has the potential to provide companies with a prolonged duration to recoup their investments and accrue revenue from their products. Moreover, governmental bodies and healthcare systems might contemplate refining reimbursement models to more accurately reflect the value of antibiotics in averting widespread resistance and sustaining effective treatments.

## 15. Strategies to Overcome Economic Challenges and Low Profitability of Antibiotic Development

**Figure 4:**
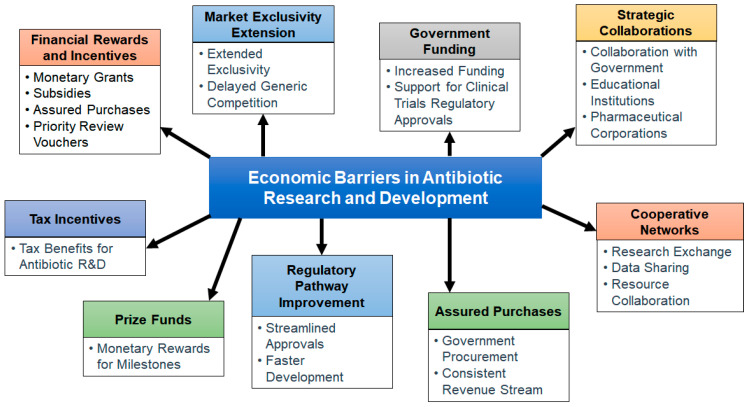
Solutions to address the economic barriers in antibiotic research and development.

To address the challenge of economic barriers and constrained profitability linked to antibiotic research and development, numerous suggested solutions and incentives have emerged (Figure 4).

Governments and public health entities have the potential to extend financial rewards or incentives to companies that effectively pioneer and introduce novel antibiotics to the market. These incentives could manifest as monetary grants, subsidies, or assured purchases, thereby securing a certain level of return on investment. Companies directing their efforts toward critical pathogens or unaddressed medical needs in the realm of antibiotics could be granted priority review vouchers. These vouchers would facilitate an expedited review process for other drugs produced by the same company.

Enlarging the timeframe of market exclusivity for recently developed antibiotics, where the introduction of generic versions is temporarily restricted, could provide companies with an extended window to recoup investments and generate revenue. Amplified government funding allocated to antibiotic research and development has the potential to alleviate some of the substantial costs associated with clinical trials and regulatory approvals.

Strategic collaborations involving governments, educational institutions, and pharmaceutical corporations can combine resources, expertise, and funding to collectively confront the challenges inherent in antibiotic development. Provision of tax incentives to companies engaged in the exploration and development of antibiotics can offer relief from the financial burdens intertwined with these endeavors.

For incentivizing breakthroughs, a prize fund established for the attainment of specific milestones in antibiotic development can serve as a catalyst, motivating companies with significant monetary rewards upon successful accomplishment [205]. International endeavors aimed at combating antibiotic resistance possess the capacity to attract funding and support from diverse sources, encompassing governmental bodies, foundations, and philanthropic organizations.

Efforts directed toward refining regulatory pathways, coupled with expediting approvals for antibiotics targeting pressing medical needs or unaddressed issues, have the potential to truncate development timelines and mitigate associated costs. The establishment of cooperative networks that facilitate the exchange of research, data, and resources can diminish duplicated efforts and elevate efficiency in the field of antibiotic development.

Governments could undertake the assurance of purchasing a predetermined quantity of newly created antibiotics, effectively creating a market and ensuring a consistent revenue stream for pharmaceutical enterprises.

## 16. Ethical and Societal Considerations

The use of antibiotics and the emergence of antibiotic resistance present complex ethical challenges within the healthcare system, while also being heavily influenced by various societal factors. The ethical considerations surrounding antibiotic use are fundamentally rooted in the principles of patient welfare, non-maleficence, and antibiotic stewardship [225]. Healthcare professionals are ethically bound to provide the best care to their patients, which includes ensuring that antibiotics are prescribed when they are necessary for treating bacterial infections. The failure to do so can result in untreated infections, patient suffering, and a breach of their ethical duty. Conversely, overprescribing antibiotics raises significant ethical concerns. It can lead to antibiotic resistance, adverse drug reactions, and disruptions to the patient’s microbiome. The ethical principle of “primum non nocere” (first, do no harm) underscores the importance of avoiding harm to patients through unnecessary interventions, including overprescribing antibiotics. Thus, the responsible use of antibiotics is an ethical obligation and reflects the concept of antibiotic stewardship, which aims to minimize resistance and protect public health.

Societal factors significantly impact antibiotic prescribing practices, often giving rise to ethical dilemmas. Overprescribing antibiotics is influenced by a fear of missing a potential bacterial infection, patient pressure to receive antibiotics, and a desire to maintain patient satisfaction [226]. Physicians may sometimes prescribe antibiotics even when there is no clear indication to do so. This pressure to meet patient expectations can compromise ethical standards. Patient expectations are a critical societal factor influencing antibiotic prescribing. Patients often anticipate receiving antibiotics when they seek medical attention. The fear of disappointing or displeasing patients can create a strong incentive for healthcare providers to prescribe antibiotics, even in cases where they may not be medically necessary. This dynamic creates a challenging ethical tension between fulfilling patient desires and adhering to the principles of responsible antibiotic use. Moreover, concerns related to liability can drive antibiotic overprescribing. Healthcare providers may prescribe antibiotics primarily to avoid potential legal repercussions. This practice, while motivated by concerns about malpractice claims, can be ethically questionable, especially if the prescription is not warranted based on clinical evidence. Public awareness campaigns, while crucial for educating the public about antibiotic resistance, have their own ethical considerations [226]. These campaigns can influence public perceptions and expectations regarding antibiotics. Therefore, it is essential that public health messages are accurate, evidence-based, and not misleading. Misinformation or messages that promote antibiotic use for minor illnesses can exacerbate the problem of overprescribing and contribute to antibiotic resistance.

## 17. Responsibilities of Healthcare Professionals

Antibiotics are potent tools in the arsenal of healthcare professionals, capable of treating bacterial infections and saving lives. However, their misuse and overuse pose ethical challenges with profound consequences. Healthcare professionals hold a central role in ensuring the judicious use of antibiotics. Their responsibilities extend beyond mere clinical expertise; they encompass ethical obligations that safeguard patient welfare and public health [227]. The ethical foundation of antibiotic use in healthcare begins with the imperative to provide the best care for patients. This duty involves a careful clinical assessment of each patient’s condition, to determine the necessity of antibiotics. Accurate diagnosis and a clear distinction between bacterial and viral infections are vital. Prescribing antibiotics without a justified medical indication breaches the fundamental ethical principle of non-maleficence, potentially exposing patients to unnecessary risks. Healthcare providers are also responsible for engaging in shared decision-making with their patients. Transparency and informed consent are key aspects of this ethical obligation. Patients must be informed about the benefits and risks of antibiotic treatment, including potential side effects and the development of antibiotic resistance [228]. This ethical communication builds trust and empowers patients to make informed decisions about their healthcare.

Antibiotic stewardship is another ethical dimension of responsible prescribing. Healthcare professionals should prescribe the narrowest-spectrum antibiotic that is effective for the patient’s condition. The appropriate dosage and duration of treatment are equally significant [229]. Stewardship necessitates the ongoing reevaluation of the need for antibiotics during treatment, with potential discontinuation if they are no longer necessary. This commitment reflects the ethical duty to prioritize patient welfare over expediency. Monitoring patients’ progress during antibiotic treatment and providing necessary follow-up care are integral components of responsible antibiotic use. Failing to fulfill these obligations may result in treatment failures, prolonged antibiotic use, and potential harm to patients. Continuous medical education is also part of the ethical landscape, as healthcare providers must stay updated on the latest guidelines, resistance patterns, and best practices for antibiotic prescribing [230].

Healthcare professionals may encounter situations where patients pressure them to prescribe antibiotics, even when they may not be medically indicated. Ethical guidelines mandate the resistance of such pressures. Healthcare providers should engage in effective communication, explain the rationale behind their decisions, and offer alternative treatment strategies when appropriate. Succumbing to patient pressure and prescribing antibiotics unnecessarily not only deviates from ethical principles, but also potentially harms patients.

## 18. Role of Policy-Makers and Regulatory Bodies

Policy-makers and regulatory bodies play a pivotal role in shaping antibiotic prescription practices, working in tandem with healthcare professionals, researchers, and the pharmaceutical industry to address the critical issue of antibiotic resistance [231]. Their responsibilities encompass a range of activities and strategies aimed at promoting responsible antibiotic use, safeguarding public health, and ensuring that antibiotics remain effective for the treatment of bacterial infections. One of the primary responsibilities of policy-makers is the development of antibiotic prescription guidelines. These guidelines are crafted in consultation with healthcare experts and practitioners, establishing evidence-based recommendations for antibiotic use. They delineate the indications, appropriate dosages, and durations of antibiotic treatment, offering healthcare providers a comprehensive framework for making informed decisions. These guidelines are a cornerstone of responsible antibiotic use, ensuring that treatment aligns with the best practices while curbing unnecessary antibiotic prescriptions. Regulatory bodies, on the other hand, oversee the availability of antibiotics. Their jurisdiction includes aspects like drug registration, distribution channels, and prescription requirements [232]. Stricter regulations enforced by these bodies can help curtail over-the-counter access to antibiotics, a significant step in reducing misuse and ensuring that healthcare professionals maintain oversight over the prescribing process.

Antibiotic stewardship programs are a collaborative effort between policy-makers and healthcare facilities. These programs are designed to optimize antibiotic use within healthcare settings. By emphasizing responsible prescription practices, continuous monitoring, and adherence to guidelines, antibiotic stewardship programs ensure that healthcare providers maintain the highest standards of care while continually evaluating the appropriateness of antibiotic prescriptions [233]. Surveillance and monitoring of antibiotic use and resistance fall under the purview of regulatory bodies. These entities collect and analyze data to identify trends and patterns, allowing for timely interventions and policy adjustments. An effective surveillance system is crucial for detecting emerging resistance and facilitating rapid responses in real-time. Educational initiatives are another essential aspect of the efforts led by policy-makers and regulatory bodies. Public awareness campaigns and educational programs serve to inform healthcare professionals and the general public about the significance of responsible antibiotic use. These initiatives raise awareness about the dire consequences of antibiotic resistance, empowering individuals to make informed health decisions [234]. Furthermore, economic incentives and penalties can be introduced by regulatory bodies to influence antibiotic prescription practices. For instance, they may provide financial rewards to healthcare facilities that adhere to antibiotic stewardship guidelines and impose fines for non-compliance. These financial measures encourage responsible prescribing. Research and development support is also a key responsibility of policy-makers. They allocate funding and resources for research into new antibiotics, diagnostics, and alternative treatments. This support is critical for addressing emerging resistance and ensuring that effective treatments remain available. Given the international nature of antibiotic resistance, policy-makers and regulatory bodies engage in international collaborations to harmonize antibiotic prescription practices, share information, and work collectively to combat resistance. This global cooperation is vital for addressing the cross-border aspect of antibiotic resistance and implementing effective measures. Lastly, policy-makers and regulatory bodies are responsible for legislating and enforcing antibiotic-related laws and regulations. They monitor and inspect healthcare facilities, pharmacies, and pharmaceutical companies to ensure compliance with these legal frameworks, thus maintaining the integrity of antibiotic prescription and use practices.

## 19. Data Availability and Reporting

The challenges in data collection and reporting of antibiotic-resistant cases pose significant hurdles to effective surveillance systems designed to combat antibiotic resistance. Addressing these challenges is essential for enhancing the quality and accuracy of data, which can, in turn, lead to improved strategies in the battle against antibiotic resistance. Several key challenges include:

(i) Under-reporting: One of the most substantial issues in surveillance systems is under-reporting. Healthcare providers and facilities may fail to report antibiotic-resistant cases due to concerns about potential consequences or the administrative burden of reporting [235]. This leads to an incomplete and inaccurate representation of the true prevalence of antibiotic resistance, hindering the ability to implement targeted interventions.
(ii) Variability in Data Quality: Data on antibiotic-resistant cases can vary significantly in quality. Variations in laboratory testing methods, diagnostic capabilities, and reporting mechanisms can lead to inconsistencies in data [236]. For instance, different facilities may use diverse criteria for defining antibiotic resistance, making it difficult to compare data across regions or healthcare systems.
(iii) Limited Access to Comprehensive Data: Access to comprehensive data is often limited, due to issues related to data sharing and privacy. Sharing patient-specific data while maintaining privacy is a delicate balance that can be challenging to achieve [237]. As a result, surveillance systems might not have access to complete patient histories, hindering the ability to track the progression of antibiotic resistance.
(iv) Heterogeneous Reporting Standards: Reporting standards and guidelines can vary significantly from one region to another. This heterogeneity can create confusion and challenges in data interpretation and integration [238]. Harmonizing these standards can streamline data collection and reporting.
(v) Data Silos: Healthcare data are often stored in isolated silos within various healthcare facilities and organizations. These silos can prevent the comprehensive sharing of data and lead to gaps in surveillance. Improved interoperability and data-sharing mechanisms are needed to facilitate data integration.
(vi) Lack of Standardization in Testing Methods: The use of varying diagnostic methods and laboratory techniques for testing antibiotic resistance can affect the quality and comparability of data. The lack of standardized testing protocols can lead to discrepancies in the reported prevalence of antibiotic resistance.
(vii) Delay in Reporting: A significant challenge is the delay in reporting antibiotic-resistant cases. The lag between data collection and reporting can hinder timely responses to outbreaks or emerging resistance patterns. Streamlining data collection, analysis, and reporting processes is crucial for faster response times.
(viii) Data Overload: The sheer volume of data generated in healthcare settings can be overwhelming, making it challenging to extract valuable information for surveillance purposes. The development of efficient data management and analysis tools is essential for identifying trends and patterns amid the data noise.
(ix) Data Security Concerns: Protecting sensitive patient data is critical, and this may limit the extent to which data can be shared among healthcare facilities and surveillance systems. Finding ways to anonymize and secure patient data while still allowing for comprehensive analysis is an ongoing challenge.
(x) Global Data Sharing: Given the global nature of antibiotic resistance, sharing data and collaborating across borders is essential. However, international data sharing can be hampered by issues such as data sovereignty, legal and ethical concerns, and differing healthcare systems.

Addressing the complex challenges in data collection and reporting of antibiotic-resistant cases is essential for strengthening surveillance systems and improving our ability to combat antibiotic resistance effectively. Several strategies can be employed to overcome these challenges:

(i) Standardizing Reporting Criteria: Establishing uniform reporting criteria and guidelines for defining and documenting antibiotic resistance is paramount. By ensuring that healthcare facilities, laboratories, and providers adhere to standardized definitions and protocols, data consistency and comparability can be significantly improved.
(ii) Improving Data Sharing Mechanisms: Enhancing data sharing mechanisms is crucial for seamless information exchange. Developing secure, interoperable systems that allow healthcare facilities and organizations to share data while safeguarding patient privacy is essential. This might involve the implementation of anonymization techniques and secure data transfer protocols.
(iii) Investing in Data Infrastructure: Investing in robust data infrastructure is fundamental for capturing, storing, and managing vast amounts of healthcare data. The development of comprehensive databases and data repositories, equipped with advanced analytical tools, can streamline data collection and analysis, making it easier to track and respond to antibiotic resistance trends.
(iv) Enhancing Data Analysis Capabilities: Effective data analysis is vital for identifying patterns, trends, and emerging resistance issues. Investing in data analysis capabilities, including the use of artificial intelligence and machine learning algorithms, can help in processing large datasets rapidly, thus enabling timely responses to changing resistance patterns.
(v) Promoting a Culture of Data Reporting and Transparency: Fostering a culture of data reporting and transparency within the healthcare community is crucial. Healthcare professionals, including clinicians and laboratory staff, need to be aware of the importance of reporting antibiotic-resistant cases. Education and awareness campaigns can play a significant role in encouraging data reporting as part of routine practice.
(vi) Collaboration and Coordination: Collaboration among healthcare facilities, public health agencies, and regulatory bodies is essential. By coordinating efforts and sharing data and insights, a more comprehensive and accurate picture of antibiotic resistance can be achieved. This collaboration can extend beyond national borders to address global antibiotic resistance challenges.
(vii) Timely Reporting: Implementing mechanisms for real-time or near-real-time reporting of antibiotic-resistant cases can significantly enhance response times. Swift reporting allows for quicker identification of outbreaks, enabling healthcare providers and policymakers to take immediate action to contain the spread of resistant strains.
(viii) Interdisciplinary Approaches: Combining expertise from various disciplines, including epidemiology, microbiology, data science, and public health, can lead to more holistic and accurate data collection and analysis. Interdisciplinary teams can work together to address the multifaceted challenges posed by antibiotic resistance.
(ix) Feedback Loops: Establishing feedback loops for healthcare facilities and providers can enhance data quality and encourage reporting. By providing facilities with timely feedback on their data, they can improve their data collection and reporting practices.
(x) Public Awareness: Educating the public about the importance of antibiotic resistance data and the role of individuals in reporting illnesses and adhering to prescribed antibiotics is crucial. A well-informed public can contribute to early reporting and more responsible antibiotic use.

## 20. International Collaborations or Initiatives Addressing Antibiotic Resistance

There are several ongoing international collaborations and initiatives aimed at addressing antibiotic resistance and promoting antibiotic research and development. These initiatives collectively contribute to a coordinated global response to the challenge of antibiotic resistance. By combining resources, expertise, and knowledge-sharing, they aim to promote responsible antibiotic use, advance research and development, and safeguard the effectiveness of antibiotics for current and future generations. While challenges persist, their efforts have contributed to a more concerted global response to this critical health threat. However, sustained commitment and collaboration are needed to ensure continued progress and effective solutions against antibiotic resistance. Some of these include:

(i) Global Antibiotic Research and Development Partnership (GARDP):

GARDP is a joint initiative of the World Health Organization (WHO) and the Drugs for Neglected Diseases initiative (DNDi). It focuses on developing new antibiotics and treatments for drug-resistant infections, particularly those affecting vulnerable populations in low- and middle-income countries [239,240]. GARDP collaborates with governments, research institutions, and pharmaceutical companies to accelerate research, development, and access to new antibiotics. GARDP focuses on bringing better solutions in two disease areas that have been greatly impacted by antibiotic resistance: serious bacterial infections that could lead to sepsis and death, with a special focus on children and newborn babies, and sexually transmitted infections such as gonorrhea.

(ii) CARB-X (Combating Antibiotic-Resistant Bacteria Biopharmaceutical Accelerator):

CARB-X is a global partnership that provides funding, expertise, and support to accelerate the development of new antibiotics, diagnostics, and other products to combat antibiotic-resistant bacteria [241]. It works with a diverse range of organizations, including academic institutions, biotechnology companies, and pharmaceutical firms, to advance innovative solutions. CARB-X has been effective in accelerating the development of innovative solutions to combat antibiotic-resistant bacteria. Several projects supported by CARB-X have advanced to preclinical and clinical stages, demonstrating its impact on facilitating progress.

(iii) Innovative Medicines Initiative (IMI) AMR Accelerator:

The IMI AMR Accelerator is a collaborative effort between the European Commission and the pharmaceutical industry. It seeks to accelerate the development of new antibiotics, diagnostics, and vaccines for bacterial infections [242]. By fostering collaboration between academia and industry, the initiative aims to address key challenges in antibiotic research and development.

(iv) ReAct—Action on Antibiotic Resistance:

ReAct is a global network of experts, researchers, and advocates working to raise awareness about antibiotic resistance and promote responsible antibiotic use [243,244]. It engages in advocacy efforts, research, and policy discussions to drive action against antibiotic resistance at local, national, and international levels. Their efforts have contributed to the global recognition of the urgent need to address antibiotic resistance.

(v) Fleming Fund:

The Fleming Fund, established by the UK government (GBP 265 million aid), focuses on strengthening surveillance systems for antimicrobial resistance in low- and middle-income countries. It provides funding and technical support to improve data collection, monitoring, and reporting of antibiotic resistance and antimicrobial use. This approach has enabled better monitoring of antibiotic resistance trends, which is essential for informed decision-making.

(vi) Global Health Security Agenda (GHSA):

GHSA is a partnership of countries, international organizations, and other stakeholders committed to preventing, detecting, and responding to infectious disease threats, including those posed by antibiotic-resistant infections. The agenda aims to enhance global health security through collaboration, capacity-building, and improved surveillance. While its impact on antibiotic resistance may not be as direct, it contributes to strengthening overall health systems and preparedness.

(vii) WHO Global Action Plan on Antimicrobial Resistance:

The World Health Organization’s Global Action Plan on Antimicrobial Resistance outlines a comprehensive strategy to address antimicrobial resistance, including antibiotic resistance. It focuses on key areas such as improving awareness, optimizing antibiotic use, and enhancing research and development for new treatments.

(viii) Joint Programming Initiative on Antimicrobial Resistance (JPIAMR):

JPIAMR is a collaborative effort among several European countries to coordinate and support research on antimicrobial resistance. It aims to fund research projects that address critical gaps in understanding antibiotic resistance and develop innovative solutions to combat it.

(ix) United Nations Interagency Coordination Group on Antimicrobial Resistance (IACG):

The IACG brings together various United Nations agencies, international organizations, and experts to provide guidance and recommendations for global efforts to combat antimicrobial resistance. It highlights the need for multi-sectoral cooperation and the development of policies that address the health, environmental, and economic aspects of antibiotic resistance.

## 21. Government-Led Initiatives against Antibiotic Resistance: A Focus on the Middle East

A global perspective on governmental efforts to combat antibiotic resistance underscores the imperative nature of this issue, which knows no borders and necessitates coordinated international action. Governments worldwide have come to acknowledge the urgent imperative of addressing antibiotic resistance, recognizing its far-reaching implications for public health, healthcare systems, economies, and environmental well-being. At the forefront of this global battle is the World Health Organization (WHO), which plays a pivotal role in orchestrating worldwide endeavors against antibiotic resistance. The WHO has devised a Global Action Plan on Antimicrobial Resistance, serving as a blueprint for nations to develop their own national action plans. In addition to providing a structured framework, the WHO initialed a program known as the Global Antimicrobial Resistance Surveillance System (GLASSS), urging its member states, including Saudi Arabia, to undertake surveillance studies on antimicrobial resistance rates on a global scale [245]. The United Nations (UN) has also identified antibiotic resistance as a threat to global health and sustainable development. This recognition has prompted high-level discussions on the international stage, underscoring the significance of concerted efforts to tackle this crisis collectively. International collaboration and partnerships have proliferated, forming a critical front in the fight against antibiotic resistance. Organizations like the Global Antibiotic Research and Development Partnership (GARDP) have emerged to accelerate the discovery and development of new antibiotics, with a particular focus on neglected and underserved populations.

Shifting the focus towards the Middle East, both Saudi Arabia and the United Arab Emirates (UAE) have emerged as proactive champions in the battle against antibiotic resistance [246]. Saudi Arabia, in particular, has demonstrated its commitment through the formulation of a comprehensive National Action Plan on Antimicrobial Resistance. This plan acts as a guiding compass, outlining a multifaceted approach to managing antibiotic resistance, encompassing surveillance, responsible antimicrobial use, infection prevention and control, and support for research and development of new antibiotics [247]. These coordinated efforts seek to bolster the Kingdom’s response to antibiotic resistance on multiple fronts. Saudi Arabia has also established robust surveillance systems to monitor antibiotic utilization and track resistance patterns. These systems collect data from healthcare facilities across the nation, offering invaluable insights into emerging resistance trends. Access to these data empowers healthcare professionals and policymakers to make well-informed decisions on antibiotic prescribing practices and interventions, enabling timely responses to outbreaks of resistance. Recognizing the pivotal role of public awareness, Saudi Arabia conducts extensive campaigns targeting both healthcare practitioners and the general populace. These initiatives educate healthcare providers on responsible antibiotic prescribing and impress upon the public the importance of using antibiotics judiciously, adhering strictly to prescribed guidelines. Further strengthening its resolve, Saudi Arabia entrusts the Saudi Food and Drug Authority (SFDA) with the critical task of regulating antibiotics. SFDA’s oversight includes stringent measures to ensure that antibiotics are prescribed, dispensed, and used judiciously, thus curbing unnecessary antibiotic consumption and mitigating the development of resistance.

The United Arab Emirates has also risen to the challenge by establishing a National Antimicrobial Resistance Committee. Comprising experts from various sectors, this committee leads the charge in crafting and implementing policies and strategies to combat antibiotic resistance. The UAE has initiated antibiotic stewardship programs within its healthcare facilities, aimed at optimizing antibiotic use. These programs closely monitor antibiotic prescriptions, promote guideline adherence, and educate healthcare providers to reduce antibiotic overuse and misuse. Recognizing the urgent need for novel antibiotics and alternative treatments, the UAE has invested significantly in research and innovation. Collaborations with international organizations and pharmaceutical firms play a pivotal role in these efforts, with the aim of contributing to the development of innovative therapies to combat antibiotic-resistant infections.

Both Saudi Arabia and the UAE actively engage in international initiatives and collaborations aimed at combating antibiotic resistance. Their commitment to aligning their strategies with global efforts underscores their dedication to addressing this pressing issue. International partnerships foster knowledge exchange, resource sharing, and collective action in the battle against antibiotic resistance. Notwithstanding their proactive efforts, Saudi Arabia and the UAE encounter challenges in their fight against antibiotic resistance. These hurdles include the need for sustained funding to support ongoing initiatives, the enhancement of data collection and reporting mechanisms, and the reinforcement of regulatory measures to ensure the responsible use of antibiotics. In the forthcoming years, both nations may explore innovative approaches to tackle these challenges. These strategies could include incentivizing pharmaceutical companies to invest in antibiotic research and development, strengthening infection prevention and control measures within healthcare settings, and further engaging the public through targeted awareness campaigns, ultimately forging a united front in the global struggle against antibiotic resistance.

## 22. Geographical Perspective of Antibiotic Resistance

Variations in antibiotic resistance patterns and the effectiveness of interventions are critical aspects of the complex global issue of antibiotic resistance. These variations occur due to a multitude of factors, including geographical location, healthcare practices, socio-economic conditions, and the implementation of intervention strategies [248]. By examining antibiotic resistance trends in specific regions or countries, the manuscript could shed light on the diverse challenges and successes faced by different healthcare systems. Geographical variation is one of the most apparent factors influencing resistance patterns. Different regions or countries can have varying resistance profiles due to variations in antibiotic usage, healthcare infrastructure, and the presence of local strains of resistant bacteria. For example, certain regions may have higher rates of antibiotic resistance due to widespread overuse or misuse of antibiotics, while others may have more stringent antibiotic stewardship programs in place. The effectiveness of interventions also varies by location. In some regions, aggressive antibiotic stewardship programs and infection control measures have led to notable reductions in antibiotic resistance. For instance, a study in Country A might show that a comprehensive stewardship program has successfully reduced the prevalence of resistant bacteria in healthcare settings. The success of interventions can be hindered by several challenges. In regions with limited resources or access to healthcare, implementing effective interventions can be a significant challenge. Even when interventions are available, compliance and adherence to guidelines by healthcare providers and patients may vary, impacting their effectiveness. Variations in local policies and healthcare practices also contribute to resistance patterns. Regions with strict regulations on antibiotic use in agriculture and aquaculture may have lower rates of antibiotic-resistant bacteria transmitted through the food chain [249]. Similarly, healthcare systems that prioritize antibiotic stewardship and infection control measures tend to have better resistance outcomes. Socio-economic factors play a significant role. Regions with high levels of poverty may face challenges in accessing healthcare and may be more prone to self-medication with antibiotics. Socio-economic status can influence the availability of healthcare resources and education on antibiotic use. Regions with well-implemented public awareness campaigns tend to see greater patient and public engagement in responsible antibiotic use. Effective educational campaigns can influence patient expectations and behaviors, reducing unnecessary antibiotic prescriptions.

In distinct geographical regions, noteworthy case studies and initiatives offer valuable insights into effective strategies for combating antibiotic resistance. One such exemplary initiative is Sweden’s “Strama” Program, an acronym for Sweden’s Strategic Program Against Antibiotic Resistance [250]. Over the years, Strama has yielded remarkable success by significantly curtailing antibiotic usage. This achievement has been realized through a multifaceted approach encompassing public education, healthcare provider guidelines, and rigorous surveillance of antibiotic prescriptions. Sweden’s dedication to Strama is reflected in its impressively low antibiotic resistance rates, positioning it as a global leader in this regard. Denmark’s “Yellow Card” initiative is another illustrative example, encouraging livestock farmers to report antibiotic usage in animals [251]. This inventive program employs a card-based system for monitoring and constraining antibiotic utilization in agriculture. This method has effectively reduced antibiotic consumption in livestock, leading to lower levels of antibiotic-resistant bacteria within food products. Scotland has adopted a targeted intervention approach aimed at mitigating *Clostridium difficile* infections within healthcare settings [252]. By conducting thorough analyses and identifying high-risk groups, the Scottish government has been able to tailor interventions precisely. The result has been a substantial reduction in the incidence of infections. The Dutch government has undertaken a comprehensive strategy through the Netherlands’ AMR Control Program to combat antibiotic resistance [253]. This program encompasses the implementation of stringent infection control measures in healthcare facilities, a reduction in antibiotic use, and substantial investments in surveillance. These collective efforts have enabled the Netherlands to maintain relatively low antibiotic resistance rates. In the United States, the Centers for Disease Control and Prevention (CDC) have played a pivotal role in advocating for antibiotic stewardship programs within healthcare settings [254]. These programs equip hospitals and clinics with invaluable guidelines and tools to optimize antibiotic usage. Certain states, such as Minnesota, have effectively implemented antibiotic stewardship initiatives that have yielded reductions in resistance rates. India stands out for its proactive stance against antibiotic resistance, epitomized by the Chennai Declaration [255]. This particular initiative emphasizes the necessity for multi-sectoral collaboration, extensive awareness campaigns, and enhanced antibiotic regulation. It underscores India’s unwavering commitment to addressing this formidable global challenge. Uganda, despite being resource-limited, has embarked on the ambitious “Uganda Research on Antibiotics Resistance and Consumption Project” (U-RESIST). This endeavor harmonizes research, data collection, and public engagement to tackle resistance head-on [256]. U-RESIST serves as a compelling exemplar of addressing antibiotic resistance in resource-constrained settings, underscoring the importance of concerted efforts in diverse global contexts.

## 23. Antibiotic Resistance in the Arabian Peninsula

The Arabian Peninsula has seen several notable case studies and initiatives that have been particularly effective in addressing antibiotic resistance. These initiatives have provided valuable insights into combating this global health challenge in the region. Saudi Arabia has taken significant steps to combat antibiotic resistance through its Saudi Arabian National Action Plan on Antimicrobial Resistance (NAPAR). The plan, developed in alignment with the World Health Organization’s Global Action Plan on Antimicrobial Resistance, outlines a comprehensive strategy for antimicrobial stewardship [257]. NAPAR focuses on improving surveillance, infection prevention and control, and promoting responsible antibiotic use in healthcare settings. The program demonstrates a commitment to addressing resistance at a national level. The United Arab Emirates (UAE) has implemented a successful Antimicrobial Stewardship Program in its healthcare facilities [258]. This initiative emphasizes rational antibiotic use, infection control, and education. By promoting judicious antibiotic prescribing and reducing the overuse of antibiotics, the UAE has made significant progress in tackling resistance in clinical settings. Kuwait launched a National Campaign for Antibiotic Stewardship to raise awareness about responsible antibiotic use among both healthcare providers and the public [259]. This campaign aimed to curb the practice of self-medication and educate the population about the risks of antibiotic resistance. By engaging the public in this manner, Kuwait has taken a proactive approach to addressing resistance. Oman has developed strong surveillance and monitoring systems to track antibiotic resistance trends. The country’s commitment to data collection and analysis has enabled healthcare professionals to make informed decisions about antibiotic use and adapt interventions as needed [260]. This data-driven approach has contributed to the success of Oman’s antibiotic resistance initiatives.

Cross-border collaboration and knowledge-sharing are essential strategies to combat antibiotic resistance effectively. In the Arabian Peninsula, where several countries share common challenges, these strategies can be particularly valuable [261]. Here are some potential approaches to foster collaboration and knowledge-sharing in the region:

(i) Establish Regional Networks: Arabian Peninsula countries can establish regional networks or consortia dedicated to antibiotic resistance research and intervention. These networks can facilitate the exchange of data, research findings, and best practices. The Gulf Cooperation Council (GCC) serves as a foundation for such collaboration, and it can be expanded to include specific working groups on antibiotic resistance.
(ii) Joint Research Projects: Collaborative research projects can be initiated to study antibiotic resistance patterns in the region. Researchers from different countries can work together to conduct multicenter studies, which will provide a more comprehensive understanding of regional challenges and help identify effective interventions. These projects can be funded by regional organizations or international research grants.
(iii) Data Sharing and Surveillance: Establishing a regional database for antibiotic resistance data could be beneficial. Participating countries can contribute data from their surveillance programs. By sharing information, countries can track resistance trends across borders and make informed decisions on interventions.
(iv) Harmonize Antibiotic Guidelines: The GCC countries have already taken steps to harmonize antibiotic guidelines. This effort can be expanded and refined to ensure consistent practices in antibiotic use, prescription policies, and resistance containment strategies. Standardized guidelines will promote effective interventions and stewardship.
(v) Regional Workshops and Conferences: Regular regional workshops and conferences can bring together healthcare professionals, researchers, and policymakers to discuss antibiotic resistance challenges and solutions. These events serve as platforms for knowledge-sharing, networking, and collaboration. They can be hosted on a rotational basis by different countries in the region.
(vi) Exchange Programs: Exchange programs for healthcare professionals can facilitate the transfer of knowledge and expertise. Doctors, pharmacists, and researchers can spend time working in healthcare institutions or laboratories in neighboring countries, gaining insights into different approaches to antibiotic resistance management.
(vii) Telemedicine and Teleconsultation: Utilize telemedicine and teleconsultation platforms to connect healthcare professionals across borders. This technology can enable experts to provide advice and guidance on complex antibiotic resistance cases, fostering cross-border cooperation.
(viii) Pharmaceutical and Industry Collaboration: Encourage pharmaceutical companies and the healthcare industry to collaborate on research and development projects targeting antibiotic resistance. Joint initiatives can lead to the development of new antibiotics and diagnostics tailored to the region’s resistance patterns.
(ix) Public Awareness Campaigns: Collaborate on public awareness campaigns that educate the public about the risks of antibiotic misuse. Shared campaigns can reach a wider audience and send consistent messages about responsible antibiotic use.

## 24. The Intersection of Artificial Intelligence (AI) and Antibiotic Resistance

AI has the potential to revolutionize how we understand, monitor, and combat antibiotic resistance. AI can play key roles in the following areas:

(a) Predictive Analytics: Predictions that are based on historical and real-time data in order to identify patterns by machine learning techniques make up predictive analytics. AI algorithms can analyze large datasets, including genetic information from bacteria, patient health records, and environmental factors, to predict which antibiotics are likely to be effective against specific strains of bacteria. This can aid clinicians in making more informed decisions about antibiotic treatment. By analyzing historical patient data, these tools can recommend the most suitable antibiotics, dosage, and duration of treatment. They can forecast patient outcomes, such as the risk of developing antibiotic resistance, mortality rates, and the length of hospital stays. This information enables medical professionals to provide more personalized and effective care. The analysis of extensive datasets on drug interactions, pharmacokinetics, and microbial genomics, and identifying potential antibiotic candidates and predicting their effectiveness can assist in development of new antibiotics. Moreover, predictive analytics is used to investigate the synergistic effects of drug combinations. This approach can uncover new treatment strategies that enhance the effectiveness of existing antibiotics and combat resistance [262].
(b) Drug Discovery: The development of antibiotics is a resource-intensive process, often requiring a long time and significant financial investments. To expedite antibiotic discovery, there is a growing need for computer-assisted exploration of innovative drugs with unique action mechanisms. Artificial intelligence (AI) has emerged as a powerful tool for accelerating antibiotic discovery. Large amounts of data can be analyzed by AI to identify novel potential drug candidates, predict their properties, and optimize their design. In this way, the drug discovery process can be quick and cheap. Virtual screening for drug discovery is one of the most promising applications of AI. Virtual screening involves using computers to simulate the interaction between potential drug candidates and bacterial targets. This can help to identify compounds that are likely to be effective against bacteria, without the need for expensive and time-consuming laboratory experiments. Another promising application of AI is drug design. AI algorithms can be used to design new antibiotics that are specifically targeted to bacterial targets [263,264].

The use of AI in antibiotic discovery is still in its early stages, but it has the capacity to alter the way we develop new antibiotics. AI has the potential to help us to identify new and effective antibiotics more quickly and efficiently, which could help to save lives in the fight against antibiotic resistance.

(c) Optimizing Treatment Plans: Artificial intelligence (AI) can optimize antibiotic treatment plans in several ways. For instance, Al can be used to identify the most effective antibiotic for a particular patient and infection. Medical history, lab results, and genetic information of the patient can be analyzed by Al, and the antibiotic that is most likely to be effective against the specific infection can be identified.

Al can be used to determine the optimal dosage and duration of antibiotic treatment for each patient. This can help to ensure that patients receive the right amount of antibiotics for the right amount of time, which can help to prevent antibiotic resistance and improve patient outcomes.

Al can be used to monitor patient data for signs of antibiotic resistance. This can help to identify patients who are at risk of developing antibiotic-resistant infections, which can then be treated with appropriate antibiotics [265,266]

(d) Surveillance and Early Detection: Al can analyze large amounts of data from clinical settings, laboratories, and public health records to identify patterns of antibiotic use and resistance. This information can be used to track the spread of resistant bacteria and inform targeted interventions. For example, Al can be used to identify hospitals or regions with high rates of antibiotic-resistant infections and target these areas for additional resources and education. Al can analyze genetic data to identify mutations that confer antibiotic resistance. This information can be used to track the evolution of resistance genes and develop new antibiotics that are less likely to be ineffective. For example, Al can be used to identify new mutations in the genes that code for antibiotic resistance in *Staphylococcus aureus*, a common cause of hospital-acquired infections. Al can develop personalized treatment plans for patients with antibiotic-resistant infections. This can be carried out by analyzing patient data, such as medical history, antibiotic use, and laboratory results. Al can also be used to identify combinations of antibiotics that are more effective against resistant bacteria [267].
(e) Rapid Diagnostics: The emergence of rapid diagnostics empowered by artificial intelligence (AI) has revolutionized the fight against antibiotic resistance. These innovative tools enable healthcare professionals to swiftly identify bacterial strains and their resistance profiles, leading to faster and more precise treatment decisions. This not only improves patient outcomes but also plays a critical role in curbing the overuse of antibiotics. Early detection of pathogens is crucial for optimal treatment of infectious diseases. Conventional methods, however, often require several days for both pathogen detection and characterization, leading to delays in initiating appropriate treatment. This delay often necessitates the use of broad-spectrum antibiotics, which can contribute to the development of AMR. Al-powered rapid diagnostics have addressed this challenge by significantly reducing the turnaround time for both detection and resistance profiling. These tools can identify specific bacterial strains and their resistance patterns within hours or even minutes, allowing clinicians to select the most effective antibiotic regimen promptly [267,268].
(f) Optimizing Antibiotic Stewardship: Antibiotic stewardship refers to the coordinated efforts to promote the responsible use of antibiotics in healthcare settings to combat antibiotic resistance, reduce unnecessary antibiotic prescriptions, and improve patient outcomes. Artificial intelligence (AI) can play a significant role in antibiotic stewardship by assisting healthcare providers and institutions in making more informed decisions about antibiotic use [267,269].

AI-powered clinical decision support systems can analyze patient data, including medical history, laboratory results, and clinical symptoms, to assist healthcare providers in making more accurate and timely decisions about whether to prescribe antibiotics. These systems can provide recommendations based on established guidelines, local resistance patterns, and the patient’s specific condition. AI can assist in determining the optimal dosage and duration of antibiotic treatment for individual patients. This can help avoid overuse or underuse of antibiotics, reducing the risk of resistance. AI can also help identify adverse effects or allergic reactions to antibiotics early, allowing for timely intervention and adjustment of treatment plans.

(g) Monitoring Environmental Factors: AI can be used to monitor environmental data in real time, which can help to identify and respond to problems quickly. Environmental data can be mapped at a high spatial resolution, which can help to identify hotspots of antibiotic contamination. Data can be analyzed from water and soil samples to detect the presence of antibiotic residues and other pharmaceutical compounds. AI can identify and track antibiotic-resistant genes by processing metagenomic data from environmental samples. Machine learning models can be developed to predict and map regions where environmental factors are more conducive to the development and spread of antibiotic resistance. Data can be analyzed from various sources to trace the origin of antibiotic contamination. AI can also analyze data related to antibiotic use in agriculture and livestock farming, thereby integrating environmental data with clinical data to better understand the links between antibiotic resistance in the environment and its impact on human health. Assisting regulatory agencies in monitoring and enforcing compliance with environmental regulations related to antibiotic use, waste management, and pollution control is yet another role for AI [266,270,271].
(h) Public Health Interventions: In the realm of public health, Artificial Intelligence (AI) plays a critical role in understanding and combating antibiotic resistance. By leveraging AI’s capabilities, extensive datasets are examined to gain insights into the dynamics of antibiotic resistance transmission, guiding the development of effective interventions. One noteworthy application of AI in the fight against antibiotic resistance is its integration with genomics for enhanced antimicrobial resistance (AMR) surveillance. AI-driven models monitor resistance genes, detect emerging trends, and identify new genetic variants, enhancing surveillance sensitivity and efficiency. These models focus on crucial features like resistance genes for initial monitoring, and continuously adapt as new data become available, ensuring accuracy. AI also integrates data from regions with high AMR transmission, facilitating ongoing monitoring and revealing correlations between AMR gene abundance and socioeconomic, health, and environmental factors. AI’s ability to identify emerging AMR genes accelerates the connection between surveillance data and patient care through diagnostic stewardship programs. This leads to updated treatment guidelines and improved patient outcomes. AI holds significant potential in the battle against antibiotic resistance and public health challenges [182].
(i) Education and Awareness: Antibiotic resistance is a growing global threat, and AI can play a crucial role in educating healthcare professionals, researchers, and the public about this critical issue. AI-driven educational tools offer several benefits in addressing antibiotic resistance. AI-powered educational tools can continuously gather and analyze the latest data on antimicrobial use and resistance, providing up-to-date information on the evolving landscape of antibiotic resistance [272]. This real-time information access ensures that healthcare providers and the public are informed about the latest trends and developments in antibiotic resistance. AI can assist researchers in analyzing vast datasets related to antibiotic resistance, helping them identify emerging resistance patterns and potential hotspots of antimicrobial resistance [262]. This ability to analyze large datasets enables researchers to gain deeper insights into the mechanisms of antibiotic resistance and inform the development of effective strategies to combat it. AI tools contribute to a broader understanding of the antibiotic resistance issue by making complex data more accessible and facilitating informed decision-making by healthcare professionals and the public. This enhanced understanding empowers individuals to make informed choices about antibiotic use and play a role in combating antibiotic resistance.
(j) Ethical Considerations: The ethical considerations of using artificial intelligence (AI) in healthcare are complex and multifaceted. There are many potential benefits to using AI in healthcare, such as improved diagnosis and treatment, but there are also risks, such as algorithmic bias and data privacy concerns. The World Health Organization (WHO) has identified six key ethical principles for the use of AI in healthcare [273,274,275]:

Protect autonomy: AI should not be used in a way that undermines patient autonomy or decision-making.

Promote human well-being: AI should be used to promote human well-being and safety, and should not be used to cause harm.

Ensure fairness and non-discrimination: the design of AI systems should be fair and its use must be non-discriminatory.

Ensure transparency: the development and use of AI should be transparent, and there should be public oversight of AI systems.

Ensure robustness and safety: AI systems should be robust and safe, and should not pose undue risks to patients or healthcare providers.

Promote responsible stewardship: AI should be used in a responsible and accountable manner, and there should be clear mechanisms for redress if AI systems are used in a way that causes harm.

Moreover, some specific ethical aspects must be considered when using AI in healthcare. The patient’s data should be collected and stored in such a way that it protects patient privacy. The algorithm on which the AI system is designed must not be discriminatory, as AI systems can be difficult to understand, which can make it difficult to hold them accountable for their decisions. AI should not be used to replace human judgment or expertise, and there should always be human oversight of AI systems. The use of AI in healthcare is an evolving field. Ethical guidelines and practices that ensure that AI is used in a safe, fair, and beneficial way for patients must continuously be developed and improved upon.

In summary, AI’s influence in the fight against antibiotic resistance is multifaceted and continually evolving. Recent research demonstrates the expanding scope of AI applications in understanding resistance mechanisms, personalized treatment strategies, proactive surveillance, and ethical considerations. By harnessing the full potential of AI and incorporating the latest developments, the healthcare sector is better equipped to combat antibiotic resistance while ensuring responsible antibiotic use.

## 25. Conclusions

The antibiotic era has witnessed a complex journey, marked by pivotal shifts in both global evolutionary and human historical contexts. The advent of antibiotics heralded a medical revolution, empowering the medical community with potent tools to combat bacterial infections. However, the widespread and often indiscriminate use of antibiotics precipitated a swift and concerning rise in antibiotic resistance, as bacteria developed mechanisms to resist the drugs’ effects. A pressing challenge in this era is the need to curtail the indiscriminate use of antibiotics, seeking more targeted approaches to address pathogens with precision. Research efforts must be dedicated to identifying potential causes of antibiotic resistance, enabling the development of early warning systems and preventive measures to preserve the efficacy of antibiotics.

The looming crisis of antibiotic resistance necessitated a fundamental shift in drug development strategies, necessitating the exploration of novel compounds, innovative drug combinations, and alternative therapeutic approaches. The profound impacts of antibiotic resistance underscore the urgency of developing new therapies capable of tackling multidrug-resistant infections. Future trends in drug development embrace the principles of precision medicine, tailoring treatments according to individual patient profiles. Leveraging cutting-edge technologies like genomics and artificial intelligence, scientists are poised to design more effective antibiotics. Collaboration among scientists, healthcare professionals, policymakers, and the pharmaceutical industry is indispensable in the battle against antibiotic resistance, ensuring long-term solutions for infectious disease management and reshaping the landscape of drug development for the years to come.

Key takeaways from the current landscape of antibiotic development and resistance underscore the urgency of addressing this global public health challenge. The dwindling antibiotic pipeline, the rise in multidrug-resistant pathogens, and the economic disincentives for pharmaceutical companies demand immediate action. The strategies include diversifying funding mechanisms, such as government incentives, to encourage investment in antibiotic research. Collaboration between academia, industry, and regulatory agencies remains pivotal for the efficient development and approval of new antibiotics. Moreover, effective stewardship programs are crucial for optimizing antibiotic use and minimizing the development of resistance.

Future research endeavors must prioritize innovative drug design, explore alternative antimicrobial approaches like phage therapy, and delve into the environmental aspects of antibiotic resistance. The adoption of the One Health approach, which acknowledges the interconnectedness of human, animal, and environmental health, will be central in tackling antibiotic resistance holistically. Finally, by harnessing the full potential of AI and incorporating the latest developments, the healthcare sector is better equipped to combat antibiotic resistance while ensuring responsible antibiotic use. As the threat of antibiotic resistance looms large, global cooperation and innovative solutions are essential to ensure the continued effectiveness of antibiotics in safeguarding public health.

## Figures and Tables

**Table 1:** Strategies to combat drug-resistant bacteria.

Strategy	Description	Examples	Ref.
New Antibiotics	Development of novel antibiotics targeting drug-resistance bacteria	Teixobactin, Lefamulin, Zoliflodacin, Cefiderocol, Eravacycline	[109,110,111]
Bacteriophage Therapy	Utilization of bacteriophages to target and kill bacteria	Phage cocktails targeting antibiotic-resistant strains	[112,113,114]
Combination Therapies	Use of multiple antibiotics in combination to enhance and prevent resistance	Trimethoprim-Sulfamethoxazole, Beta-lactam/beta-lactamase inhibitor combinations	[115,116]
Immune Modulation	Boosting the immune response to enhance clearance of bacterial infections	Interferons, interleukins, colony-stimulating factors, etc.	[117]
Antibody Therapies	Development of monoclonal antibodies or antibody-derived molecules to target bacterial pathogens	Bezlotoxumab, altastaph, lysibodies, antibody-toxin conjugates	[118]
Nanotechnology	Use of nanoparticles for drug delivery or disruption of bacterial membranes	Metal–organic frameworks, carbon quantum dots	[119,120]
Heterocyclic compounds	Use of heterocyclic compounds as novel antimicrobial agents	Thiazole, Imidazole, Thiazolidinone, Oxazole, Pyrrole, Pyridine, Pyrimidine	[121]
Antimicrobial Peptides	Exploration of naturally occurring peptides with antimicrobial properties	LL-37, defensins, magainins, temporins, melittin	[122,123,124]
CRISPR-Cas Systems	Using CRISPR gene-editing technology to target and eliminate drug-resistant bacteria	CRISPR-Cas systems for selective targeting of bacterial DNA	[125,126,127]
One Health Approach	Holistic approach integrating human health, animal health, and the environment	Coordinated efforts to combat antimicrobial resistance across various sectors	[128,129]
Drug Repurposing	Investigating existing drugs for potential activity against drug-resistant microorganisms	Statins, chlorpromazine, thioridazine	[130,131]
Surveillance and Diagnostics	Improved monitoring and diagnostic tools for early detection and tracking of resistance	PCR, next-generation sequencing, telemedicine, biosensors, microfluidic devices	[132,133,134]

**Table 2:** Safety, efficacy, and commercial viability of some common alternative therapies (compared with traditional antibiotics) against antibiotic resistance.

Strategies	Safety	Efficacy	Commercial Viability	Ref.
Antibiotics	Traditional antibiotics can have a broad spectrum of activity, affecting both harmful and beneficial bacteria in the body. This can lead to disruptions in the microbiome and potential side effects.	Traditional antibiotics have a history of widespread use and can be effective against a wide range of bacterial infections. However, efficacy can decrease due to resistance.	Traditional antibiotics have a well-established market and regulatory pathways. However, increased resistance, lengthy development times, and low profit margins have reduced their commercial viability.	[189]
Antimicrobial peptides	Generally safe; Minimal toxicity; Concerns in high doses.	Effective against some bacterial strains; Ongoing development	Potential commercial viability with research and development.	[73]
Monoclonal antibodies	Highly safe profile; Minimal side effects.	Effective in targeting specific pathogens; Reduce infection risks.	Costly research and development; Potential regulatory approval challenges.	[190]
Probiotics	Generally safe; Risk in individuals with compromised immune systems.	Effective for certain infections; Efficacy varies by strain, infection type, and host factors.	Gained commercial traction in the dietary supplement and functional food industries; Need more investment for specific bacterial infections.	[191]
Bacteriophages	Generally safe; Minimal side effects.	Variable, efficacy may be phage-specific.	Potential viability; Ongoing research.	[192]
Phage therapy	Generally safe; Minimal side effects.	Variable, depending on specificity, research and matching phages.	Increasing interest and investment with ongoing studies.	[193]
Essential oils and plant extracts	Generally safe.	Challenges in standardization of their components.	Potential viability; Ongoing research.	[194]
Nanoparticles	Long-term use needs evaluation.	Effective against some bacterial strains; Ongoing development.	High-quality nanoparticle production can be costly.	[171]

## Data Availability

Data sharing is not applicable.
